# Traditional Chinese exercise for quality of life, cognition, sleep in Parkinson’s disease: a systematic review and meta-analysis

**DOI:** 10.3389/fpsyg.2026.1824910

**Published:** 2026-06-09

**Authors:** Qing Yan, Zhe Bai, Zejing Sha, Keying Zhang, Dongxue Liang, Shanjun Li, Dong Zhang

**Affiliations:** 1 Institute of Artificial Intelligence in Sports, Capital University of Physical Education and Sports, Beijing, China; 2 Department of Physical Education, Southeast University, Nanjing, China

**Keywords:** cognition, neuropsychiatric, Parkinson’s disease, quality of life, sleep, traditional Chinese exercises

## Abstract

**Objective:**

This review aims to synthesize the current evidence on the potential effects of Traditional Chinese Exercise (TCE) on cognition, sleep quality, and quality of life (QoL) in Parkinson’s disease (PD) patients, and to explore intervention characteristics that may be associated with beneficial outcomes.

**Methods:**

We searched five major databases (PubMed, Web of Science, EBSCO, Cochrane, and Scopus) for randomized controlled trials up to March 2025. Studies assessing TCE in patients with PD were included. Data were pooled using the standardized mean difference (SMD) and 95% confidence interval (CI).

**Results:**

Fifteen trials met the inclusion criteria. TCE demonstrated significant overall improvements in cognition (SMD = 0.87, 95% CI [0.56, 1.19]), sleep quality (SMD, −0.88, 95% CI [−1.41, −0.34]), and QoL (SMD, −0.48, 95% CI [−0.66, −0.29]). Subgroup analyses revealed that interventions lasting ≥12 weeks and totaling ≥180 min per week may be associated with more favorable outcomes, although these findings should be interpreted cautiously. For exercise type, Tai Chi was particularly effective for cognition (SMD, 0.91, *p* < 0.0001) and QoL (SMD, −0.70, *p* < 0.00001), while Health Qigong was highly effective for sleep quality (SMD, −0.92, *p* = 0.01). Benefits were also more pronounced in patients with a disease duration of 5 years or more.

**Conclusion:**

TCE may be beneficial for improving cognition, sleep quality, and QoL in patients with PD. Subgroup findings further suggest that intervention type, duration, and weekly exercise volume may be associated with differential outcomes.

## 1 Introduction

Parkinson’s disease (PD), the second most prevalent neurodegenerative disorder after Alzheimer’s disease, is characterized primarily by motor symptoms, including bradykinesia, myoclonus, and postural instability (Sungkarat et al., 2018; Ben-Shlomo et al., 2024). In addition to these hallmark motor manifestations, PD is frequently accompanied by a range of non-motor symptoms such as sleep disturbances, and cognitive impairment. PD represents an increasingly significant global health burden, with an estimated 11.9 million people affected worldwide in 2021. This figure is projected to increase substantially to 25.2 million by 2050, primarily due to population aging and growth. The typical age of onset for PD is between 50 and 60 years. The prevalence increases with age, reaching 1.7% among individuals over 65 years of age and exceeding 4% among those over 80 (Sungkarat et al., 2018). Notably, non-motor symptoms (NMS), including insomnia, constipation, depression, anxiety, and cognitive decline, affect up to 56% of patients even in early, untreated PD stages (Goldman and Postuma, 2014; Tolosa et al., 2021).

Although drugs such as Levodopa are established as standard therapy for the motor symptoms of Parkinson ‘s disease (de Boer et al., 1996; Snell et al., 2016; Peng et al., 2024), their effectiveness in alleviating non-motor symptoms is limited (Kulisevsky, 2000; Molloy et al., 2006), This is presumably due to the fact that many non-motor symptoms are related to dysfunction of non-dopaminergic nervous Systems such as the cholinergic, serotonergic, and noradrenergic Systems (Schapira et al., 2017). In addition, research on the effects of Levodopa on cognitive function is inconsistent, and discussion continues (Kulisevsky, 2000; Molloy et al., 2006). Against this background, the importance of non-pharmacological approaches in addition to drug therapy in the treatment of non-motor symptoms of Parkinson’s disease has been increasingly recognized in recent years.

Exercise therapy serves as an effective adjunct to levodopa treatment. Physical activity helps to slow the decline of motor skills. It also allows PD to remain functionally independent for longer (Goodwin et al., 2008; Tomlinson et al., 2013). Traditional Chinese exercise (TCE), primarily encompassing Tai Chi and Health Qigong, emphasizes slow, gentle, and symmetrical movements, meditative states, and breath control, integrating physical activity with traditional health philosophies to promote physical and mental well-being. These exercises are primarily of low to medium intensity, which is suitable for Parkinson’s patients, especially for middle-aged and elderly people (Li et al., 2025; Tan et al., 2025). Accumulating evidence suggests TCE benefits both motor and non-motor symptoms in PD, such as balance, gait, postural stability, and sleep (Wu et al., 2011; Ernst et al., 2024; Li et al., 2025). A growing body of evidence supports these benefits. Although several systematic reviews have evaluated the effects of traditional Chinese exercise in patients with Parkinson’s disease, some limitations remain in how Health Qigong interventions have been represented. In certain previous studies, Qigong was searched for or classified mainly at a broad category level, which may not have fully reflected the range of specific Health Qigong forms. This may have limited a more comprehensive understanding of the overall effects of traditional Chinese exercise. Moreover, systematic evidence regarding the effects of traditional Chinese exercise on non-motor symptoms in Parkinson’s disease across different intervention durations, exercise volumes, and patient disease durations remains limited. In particular, factors that may influence treatment response, such as disease duration, have been less frequently examined in previous evidence syntheses.

Randomized controlled trials (RCTs) are widely recognized as the gold standard for generating high-quality scientific evidence. In this meta-analysis of RCTs, we evaluated the current evidence on TCE interventions in reducing cognition, SD, QoL in individuals with PD. This review aims to provide an updated synthesis of the latest scientific evidence up to 2025 scientific evidence regarding the therapeutic effects of TCE for to inform future research and clinical investigation.

## 2 Methods

### 2.1 Search strategy

For this study, we conducted a comprehensive search of five major electronic databases—PubMed, Web of Science, EBSCO, Cochrane, and Scopus. Search terms were developed using Medical Subject Headings (MeSH) and free-text keywords, combined using Boolean operators to balance sensitivity and specificity. The final search string applied across databases was: (‘Parkinson’s disease’) AND (‘Tai Ji’ OR ‘Qigong’ OR ‘Ba Duan Jin’ OR ‘Wu Qin Xi’ OR ‘Yi Jin Jing’ OR ‘Liu Zi Jue’ OR ‘Traditional exercises’). The search was conducted from January 1, 2010 to March 9, 2025. No methodological filters were applied in the search strategy to guarantee high recall; eligible randomized controlled trials were subsequently identified during the literature screening process according to the prespecified inclusion criteria. The search strategies for electronic databases are listed in Supplementary Table 1. Additionally, we manually reviewed the reference lists of all identified systematic reviews and meta-analyses to identify any additional relevant studies. The entire process was independently completed by two authors (QY and ZB) following a standardized procedure. In cases of disagreement, a third author (SZJ) participated in the discussion until a consensus was reached among all three authors. This review was conducted in accordance with PRISMA guidelines. The review protocol was not prospectively registered.

### 2.2 Study selection: inclusion and exclusion criteria

The inclusion criteria for this study were as follows: (1)study type: randomized controlled trials (RCTs) design reported in English; (2) intervention group: TCE include various styles of Tai Chi and Health Qigong, such as Tai Chi, Qigong, Ba Duan Jin, Wu Qin Xi, Liu Zi Jue, Yi Jin Jing; (3) control groups: routine treatments, no intervention, sham TCE; (4) participant type: individuals with PD under clinical diagnosis;(5) outcomes: Assessed at least one of the following: cognitive function, sleep quality, depressive symptoms, anxiety symptoms, or health-related QoL.

Exclusion criteria: (1) animal studies, review articles, conference papers, case reports, meta-analyses; (2) interventions unrelated to the research topic; (3) non-RCT studies; (4) studies where the full text is unavailable or data are missing.

### 2.3 Quality assessment and data extraction

The risk of bias was assessed by the author (ZB) using the Cochrane Collaboration’s risk-of-bias tool. Given the nature of exercise interventions, blinding participants in the included studies is impractical. Thus, only six other types of bias risk were evaluated, including random sequence generation, allocation concealment, blinding of outcome assessors, incomplete outcome data, selective outcome reporting, and other risks of bias. Each domain was judged to be low, unclear, or high risk of bias. The final assessment for all studies was presented in a “risk of bias” table. Data extraction was independently conducted by two authors, which encompassed the following: (a) characteristics of the included studies (first author’s last name, publication year, sample size); (b) intervention characteristics (type of intervention, intervention duration, frequency, session duration, weekly time); (c) participant characteristics (disease duration, age, sex); (d) outcome data: mean and standard deviation (SD) for pre- and post-intervention values, or change scores, for the relevant non-motor outcomes.

### 2.4 Statistical analysis

The outcomes in this article are all continuous variables. To unify the pooling of all studies, where some employed identical scales but reported outcomes differently (e.g., mean ± standard deviation versus change scores), we selected SMD as the primary effect measure. RevMan 5.3 software was used to assess the risk of bias and to conduct traditional meta-analyses. The *I^2^* statistic was employed to evaluate the magnitude of heterogeneity. Sensitivity analyses were conducted by sequentially excluding individual studies to assess the stability of the results; any exclusion was solely for sensitivity testing and did not serve as the primary analytical strategy. According to the Cochrane guidelines, heterogeneity was interpreted as follows: *I^2^* = 25% (low heterogeneity), *I^2^* = 50% (moderate heterogeneity), and *I^2^* = 75% (high heterogeneity; Bai et al., 2025). Studies contributing to the high heterogeneity were excluded. A funnel plot was used to assess publication bias after determining the overall effect size. When *I*^2^ > 50%, it indicates significant heterogeneity among the included studies. In such cases, subgroup analysis was performed to identify potential sources of heterogeneity. If the heterogeneity could not be reduced, a random-effects model was applied, and results were interpreted descriptively. To investigate potential sources of heterogeneity, we conducted subgroup analyses based on several *a priori*, evidence-based criteria. First, we stratified studies according to the guidelines of the American College of Sports Medicine (ACSM; Bayles, 2023). These guidelines suggest a commonly used neuromotor training frequency of at least 2–3 times per week 30–60 min per seeion. Accordingly, we categorized intervention frequency as < 180 min per week versus ≥ 180 min/week. Second, as it is well-established that a duration of at least 12 weeks is required for stable improvements in non-motor symptoms such as cognitive function (NORTH et al., 1990; Northey et al., 2018), we stratified the total intervention period into short-term (< 12 weeks) and long-term (≥ 12 weeks). Finally, in accordance with the clinical criteria from the International Parkinson and Movement Disorder Society (MDS), we grouped studies by disease duration into early-stage (< 5 years) and mid-to-late stage (≥ 5 years; Postuma et al., 2015). Forest plots were generated using RevMan 5.3, while meta-regression and funnel plots were performed using Stata software. Statistically significant outcomes were considered at *p* < 0.05.

## 3 Results

### 3.1 Study selection

**Figure 1:**
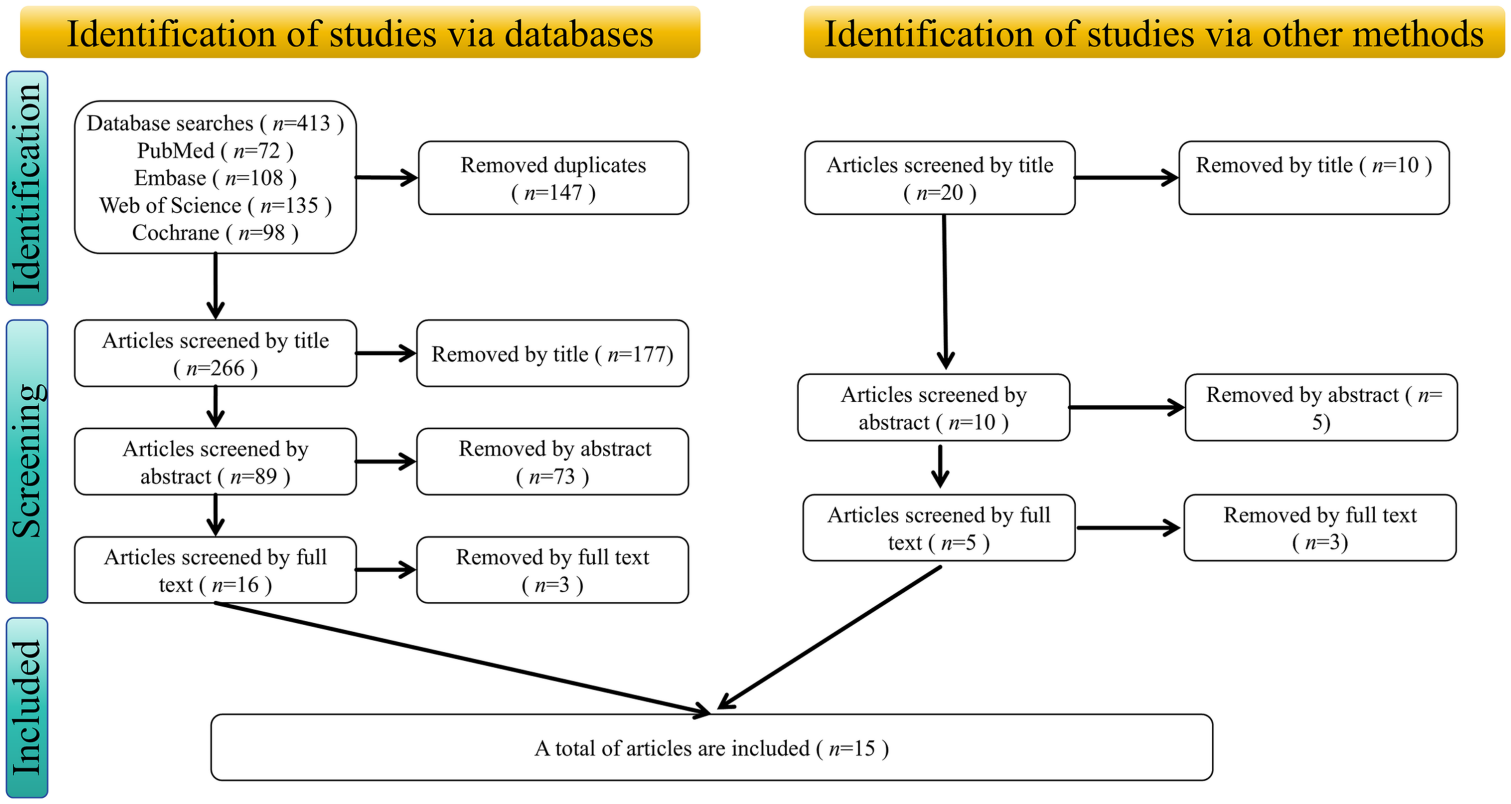
Flow diagram of studies included in the review.

Figure 1 illustrates the initial retrieval of 413 records from the databases, and 20 records were identified from other sources. After removing duplicates, 286 studies remained. After screening the titles and abstracts, 21 potentially eligible studies were identified. After full-text assessment, 6 studies were excluded because they did not meet the inclusion criteria. Ultimately, 15 studies met the inclusion criteria.

### 3.2 Characteristics of included

The included studies were conducted in 4 countries, the most common countries being China (9 trials) and the USA (4 trials; Li et al., 2014; Xiao and Zhuang, 2016; Zhu et al., 2020; Li X. et al., 2022; Wang Z. et al., 2022; Chang et al., 2024; Li et al., 2024; Tsai et al., 2025; Yin et al., 2025; Nocera et al., 2013; Moon et al., 2017; Vergara-Diaz et al., 2018; Moon et al., 2020). The remaining studies were carried out in Germany (1 trial) and Korea (1 trial; Poier et al., 2019; Cheon et al., 2013). The studies included a total of 656 PD participants, with disease duration ranging from 2.9 to 12.6 years across the patient population. The studies included a total of 656 PD participants, with disease duration ranging from 2.9 to 12.6 years across the patient population. In terms of focus, 5 studies targeted cognition (Moon et al., 2020; Zhu et al., 2020; Wang Z. et al., 2022; Chang et al., 2024; Tsai et al., 2025), 5 studies addressed sleep quality (Xiao and Zhuang, 2016; Moon et al., 2017; Moon et al., 2020; Zhu et al., 2020; Wang Z. et al., 2022), and 11 studies assessed quality of life (Cheon et al., 2013; Nocera et al., 2013; Li et al., 2014; Vergara-Diaz et al., 2018; Poier et al., 2019; Moon et al., 2020; Zhu et al., 2020; Li Z. L. et al., 2022; Wang Z. et al., 2022; Li et al., 2024; Yin et al., 2025). These studies encompassed four types of 9 Health Qigong exercises: 2 Qigong (Moon et al., 2017; Moon et al., 2020), 1 Liu Zi Jue (Yin et al., 2025), 2 Ba Duan Jin (Xiao and Zhuang, 2016; Li et al., 2024), 2 Wu Qin Qi (Li Z. L. et al., 2022; Wang Z. et al., 2022), as well as 8 Tai Chi exercises (Cheon et al., 2013; Nocera et al., 2013; Li et al., 2014; Vergara-Diaz et al., 2018; Poier et al., 2019; Zhu et al., 2020; Chang et al., 2024; Tsai et al., 2025). The frequency of weekly interventions ranged from 1 to 4 times per week. The session duration ranged from 15 to 90 min. Finally, the weekly time spent on interventions ranged from 60 to 300 min. More detailed information about the included studies can be found in Table 1.

**Table 1:** Characteristics of the RCTs included in the systematic review.

Author, year (country)	Sample size		Age, years		Gender (M: F)		Disease course		Interventions		Frequency	Outcome measures
	T	C	T	C	T	C	T	C	T	C		
Li et al. (2024) (China)	27	27	65.59 ± 9.16	60.48 ± 11.52	3:14	10:17	4.59 ± 3.70	7.56 ± 7.95	Health Qigong (Baduanjin)	Routine exercise	5 times/week, 40 min/session, 4 weeks	PDQ-39
Zhu et al. (2020) (China)	19	22	68.53 ± 1.90	67.77 ± 1.72	12:7	13:9	4.68 ± 0.43	4.00 ± 0.39	Tai Chi + Routine exercise	Routine exercise	3 times/week, 40–50 min/session, 12 weeks	HAMD, HAMA, MoCA, PDQ-39, PDSS
Yin et al. (2025) (China)	25	26	58.80 ± 8.61	58.15 ± 7.09	12:13	12:14	6.05 ± 3.07	6.15 ± 4.47	Health Qigong (Liuzijue)	Routine exercise	5 times/week, 30 min/session, 12 weeks	HAMD, HAMA, PDQ-39
Li et al. (2014) (China)	65	65	68.7 ± 8.7	68.7 ± 8.7	NR	NR	NR	NR	Tai Chi	Routine exercise	2 times/week, 60 min/session, 6 months	PDQ-8
Moon et al. (2020) (USA)	8	9	66.4 ± 8.10	65.9 ± 5.40	4:4	3:6	4.25 ± 2.10	5.33 ± 3.30	Health Qigong	Sham Health Qigong	15–20 min twice a day + 45 min1h of group exercise/week, 12 weeks	PDSS− 2, GAI, GDS, MMSE, PDQ
Moon et al. (2017) (USA)	5	5	61.8 ± 5.7	68.0 ± 5.3	NR	NR	8.0 ± 3.6	13.3 ± 3.6	Health Qigong	Sham Health Qigong	15–20 min twice a day, 6 weeks	PDSS-2
Nocera et al. (2013) (USA)	15	6	66.0 ± 11.00	65.00 ± 7.00	7:8	4:2	8.08 ± 5.42	6.83 ± 1.83	Tai Chi	No intervention	3 times/week, 60 min/session 16 weeks	PDQ-39
Vergara-Diaz et al. (2018) (USA)	16	16	65.7 ± 3.9	62.0 ± 7.8	9:7	7:9	2.9 ± 2.4	2.9 ± 2.2	Tai Chi	No intervention	2 times/week, 60 min/session, 6 months	PDQ-39
Li Z. L. et al. (2022) (China)	20	20	67.57 ± 3.95	70.00 ± 5.59	13:7	16:4	6.83 ± 4.09	7.76 ± 4.55	Health Qigong (Wuqinxi)	Routine exercise	2 times/week, 90 min/session, 12 weeks	PDQ-39
Wang Z. et al. (2022) (China)	23	22	68.83 ± 4.35	67.95 ± 4.86	7:16	10:12	6.63 ± 4.01	6.09 ± 3.85	Health Qigong (Wuqinxi)	Routine exercise	3 times/week 90 min/session, 24 weeks	HADS, PDSS, MOCA, PDQ-39
Xiao and Zhuang (2016) (China)	50	50	67.53 ± 8.56	66.52 ± 2.13	NR	NR	5.45 ± 3.61	6.15 ± 2.63	Health Qigong (Baduanjin)	Routine exercise	4 times/week, 45 min/session, 6 months + daily walking 30 min	PDSS-2
Poier et al. (2019) (Germany)	15	14	68.9 ± 11.0	68.5 ± 8.1	3:12	9:5	NR	NR	Tai Chi	Tango	1 time/week, 60 min/session, 10 weeks	PDQ-39
Cheon et al. (2013) (Korea)	9	7	65.6 ± 7.9	64.9 ± 7.2	All female	All female	6.1 ± 2.9	4.7 ± 4.2	Tai Chi	No intervention	3 times/week, 50–60 min/session, 8 weeks	PD-QoL, BDI
Chang et al. (2024) (China)	16	13	66.31 ± 6.54	63.15 ± 7.95	7:9	6:7	6.75 ± 5.49	6.77 ± 6.84	Tai Chi	Routine exercise	2 times/week, 60 min/session, 12 weeks	MOCA, BDI-II
,Tsai et al. (2025) (China)	21	20	64.67 ± 8.05	67.00 ± 7.07	13:8	11:9	12.62 ± 4.14	6.10 ± 4.03	Tai Chi	Routine exercise	2 times/week, 60 min/session, 12 weeks	MMSE, BDI-II

HAMD, Hamilton Depression Rating Scale; BDI, Beck Depression Inventory; MADRS, Montgomery–Åsberg Depression Rating Scale; GDS, Geriatric Depression Scale; PDSS-2, Parkinson’s Disease Sleep Scale-2; PDSS, Parkinson’s Disease Sleep Scale; PDQ-39, Parkinson’s Disease Questionnaire-39; PDQ-8, Parkinson’s Disease Questionnaire; PD-QoL, Parkinson’s Disease QoL; HDRS-17, Hamilton Depression Rating Scale-17.

### 3.3 Study quality assessment

The Cochrane Risk Assessment Tool was utilized to assess the methodological quality of the included studies, which focuses on six biases: selection, performance, detection, attrition, reporting, and others. As shown in the Supplementary Table 1. The included studies were categorized into three quality levels—low, medium, and high—based on specific methodological criteria. Studies were rated as low quality if they exhibited a high risk of bias in random sequence generation or allocation concealment, even if other sources of bias were also present. High-quality studies were defined as those with a low risk of bias in both randomization and allocation concealment, and with all other domains assessed as either low or unclear risk. Studies that did not meet the criteria for either high or low quality were classified as medium quality. We applied a widely used methodological quality assessment tool to assess the methodological rigor of individual studies. Of the 15 included trials, 6 rated as high quality, 6 as moderate quality, and 3 as low quality (Higgins et al., 2011) (Supplementary Figure 1).

### 3.4 Meta-analysis result

#### 3.4.1 Primary outcomes

- Quality of life

**Figure 2:**
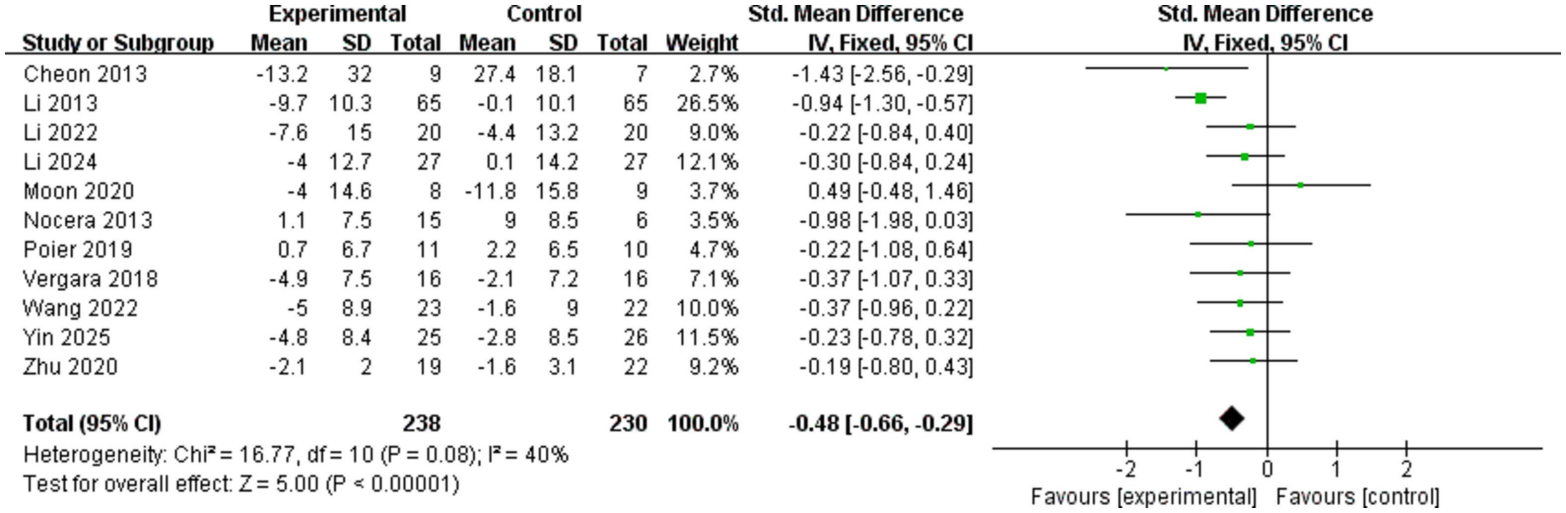
Forest plot of Parkinson’s QoL.

11 studies reported data on QoL, encompassing 238 participants in the experimental groups and 230 in the control groups. Compared with the control condition, TCE demonstrated a significant positive effect on QoL in PD patients [SMD = −0.48, 95% CI (−0.66–−0.29), *p* < 0.00001, *I^2^* = 40%; low heterogeneity; Figure 2]. To investigate the influence of key moderators and to assess the stability of our findings, we performed subgroup and meta-regression analysis and sensitivity analyses.

Subgroup analyses data revealed that Tai Chi (SMD: −0.70, 95% CI: −0.95– −0.44, *p* < 0.00001), a total weekly duration of ≥ 180 min (SMD: −0.39, 95% CI: −0.65– −0.14, *p* = 0.003), as well as both intervention durations (<12 weeks: SMD: −0.43, 95% CI: −0.86– −0.01, *p* = 0.04; ≥12 weeks: SMD: −0.49, 95% CI: −0.69– −0.28, *p* < 0.00001) had statistically significant effects on QoL, with the longer duration showing a slightly stronger effect. Furthermore, subgroup analysis also revealed that only significantly improved QoL with a disease duration of ≥ 5 years (SMD: −0.56, 95% CI: −0.77– −0.35, *p* < 0.00001; Supplementary Figure 2; Table 2).

**Table 2:** Subgroup analysis results of QoL.

Subgroup		Number of references	Heterogeneity test	Results of meta-analysis	*p*
			*I^2^*	SMD (95%CI)	
Interventions	Tai Chi	6	39%	-0.70[−0.95, −0.44]	<0.00001
	Health Qigong	5	0%	−0.22[−0.50, −0.05]	0.11
Disease duration	≥ 5 years	8	38%	−0.56[−0.77, −0.35]	<0.00001
	< 5 years	3	2%	−0.13[−0.54, 0.29]	0.55
Intervention duration	<12 weeks	3	41%	−0.43[−0.86, −0.01]	0.04
	≥ 12 weeks	8	48%	−0.49[−0.69, −0.28]	<0.00001
Weekly time	≥ 180 min	7	0%	−0.39[−0.65, −0.14]	0.003
	< 180 min	4	72%	−0.32[−0.92, 0.27]	0.28

#### 3.4.2 Secondary outcomes

- Cognitive function

**Figure 3:**
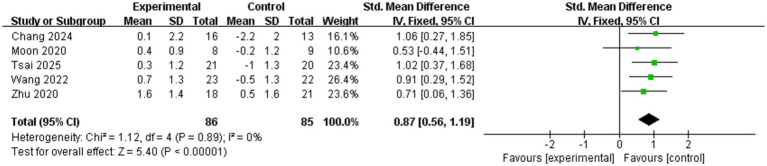
Forest plot of the cognition.

Five studies reported data on cognitive outcomes, encompassing 86 participants in the experimental groups and 85 in the control groups. Compared with the control condition, TCE demonstrated a significant positive effect on cognitive function in PD patients [SMD = 0.87, 95% CI (0.56–1.19), *p* < 0.00001, *I^2^* = 0%; Figure 3]. The *I^2^* statistic suggested low statistical heterogeneity. However, it is important to note that when the number of included studies is small, I^2^ has limited power to detect true heterogeneity; therefore, a low I^2^ value does not necessarily indicate complete homogeneity across studies. To address potential sources of clinical and methodological variability, we conducted additional subgroup analyses despite the low *I^2^* value. Subgroup analyses revealed that Tai Chi (SMD: 0.91, 95% CI: 0.52–1.31, *p* < 0.00001), a total weekly duration of ≥ 180 min (SMD: 0.91, 95% CI: 0.29–1.52, *p* = 0.004), and an intervention duration of ≥ 12 weeks (SMD: 0.70, 95% CI: 0.40–1.11, *p* < 0.00001) showed the strongest effects on cognition. Furthermore, subgroup analysis also revealed significantly improved cognitive function in patients with a disease duration of ≥ 5 years (SMD: 0.96, 95% CI: 0.57–1.35, *p* < 0.00001; Supplementary Figure 3; Table 3).

- Quality of Sleep

**Table 3:** Subgroup analysis results of cognition.

Subgroup		Number of references	Heterogeneity test	Results of meta-analysis	*p*
			*I^2^*	SMD (95%CI)	
Interventions	Tai Chi	3	0%	0.91[0.52, 1.31]	<0.00001
	Health Qigong	2	0%	0.80[0.28, 1.32]	0.003
Disease duration	≥ 5 years	3	0%	0.96[0.57, 1.35]	<0.00001
	< 5 years	2	0%	0.66[0.12, 1.20]	0.02
Intervention duration	<12 weeks	No studies	-	-	-
	≥ 12 weeks	5	45%	0.70[0.40, 1.11]	<0.0001
Weekly time	≥ 180 min	1	-	0.91[0.29, 1.52]	0.004
	< 180 min	4	0%	0.86[0.49, 1.23]	<0.0001

**Figure 4:**
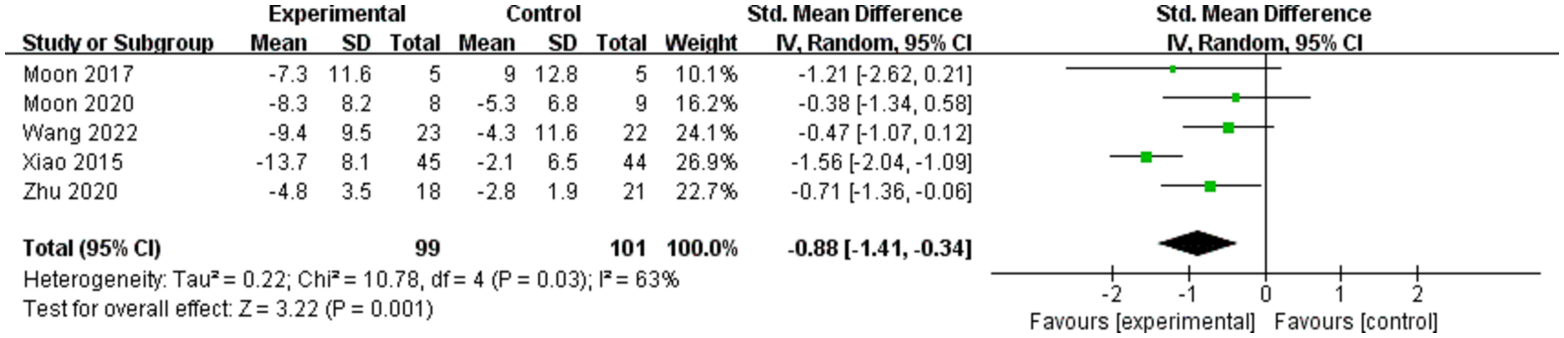
Forest plot of Parkinson’s disease sleep.

Five studies reported data on sleep quality, encompassing 99 participants in the experimental groups and 101 in the control groups. Compared with the control condition, TCE demonstrated a significant positive effect on sleep disorders in PD patients [SMD = −0.88, 95% CI (−1.41–−0.34), *p* = 0.001, *I^2^* = 63%; moderate heterogeneity; Figure 4]. Subgroup analyses data revealed that only a total weekly duration of ≥ 180 min (SMD: −0.94, 95% CI: −1.46–−0.24, *p* = 0.009) and ≥ 12 weeks (SMD: −0.84, 95% CI: −1.44–−0.23, *p* = 0.007) showed effects on sleep quality. Furthermore, subgroup analysis also revealed that Health Qigong (SMD: −0.92, 95% CI: −1.62–−0.06, *p* = 0.03) and a disease duration of ≥ 5 years (SMD: −1.07, 95% CI: −1.90–−0.25, *p* = 0.01) showed the best effects on sleep quality (Supplementary Figure 4, Table 4).

**Table 4:** Subgroup analysis results of sleep quality.

Subgroup		Number of references	Heterogeneity test	Results of meta-analysis	*p*
			*I^2^*	SMD (95%CI)	
Interventions	Tai Chi	1	-	−0.71[−1.36, −0.06]	-
	Health Qigong	4	70%	−0.92[−1.62, −0.22]	0.01
Disease duration	≥ 5 years	3	75%	−1.07[−1.90, −0.25]	0.01
	< 5 years	2	0%	−0.61[−1.15, −0.07]	0.03
Intervention duration	<12 weeks	1	-	−1.21[−2.62, −0.21]	0.09
	≥ 12 weeks	4	72%	−0.84[−1.44, −0.23]	0.007
Weekly time	≥ 180 min	3	78%	−0.94[−1.46, −0.24]	0.009
	< 180 min	2	0%	−0.64[−1.44, 0.15]	0.11

### 3.5 Regression analysis

To explore potential sources of heterogeneity in the outcomes related to QoL, cognitive function and sleep quality, meta-regression analyses were conducted focusing on participant characteristics, specifically age and disease duration. The results indicated no statistically significant associations for either outcome. For QoL, neither age (*p* = 0.698) nor disease duration (*p* = 0.297) showed notable effects. Similarly, for cognitive function, neither age (*p* = 1.000) nor disease duration (*p* = 1.000) demonstrated a significant association with the pooled effects. In the domain of sleep quality, age (*p* = 0.708) and disease duration (*p* = 0.648) were also not found to exert significant influence. (Supplementary Figure 5; Table 5). The lack of significant findings may be partly attributed to the relatively small number of included studies in each subgroup. Therefore, these analyses should be regarded as exploratory. Meta-regression was not performed for cognitive function or sleep quality due to the insufficient number of included studies (fewer than 10 per outcome), which would have yielded underpowered and potentially unreliable estimates.

**Table 5:** Summaries of meta-regression analyses.

Outcome measure	Predictor	Meta-regression coefficient	Standard error	95%CI	*p*-value
QoL	Duration of illness	−0.936	0.089	[−0.269, 0.082]	0.297
	Age	−0.014	0.038	[−0.09, 0.06]	0.698
Cognition	Duration of illness	0	0.083	[−0.163, 0.163]	1
	Age	0	6.929	[−0.203, 0.203]	1
Sleep quality	Duration of illness	−0.069	0.151	[−0.366, 0.228]	0.648
	Age	0.09	0.242	[−38.905, –24.965]	0.708

### 3.6 Publication bias

To determine if there is any bias in the publications, we conducted the funnel plot for outcome (QoL) indicators with≥ 10 included studies. The funnel plot shows a slight asymmetry, with two scattered studies located at the bottom of the funnel plot, indicating a possible small sample effect. It is worth noting that the effect estimates of the included studies mainly contain non-significant values (*p* > 10%), indicating that the asymmetry of the funnel plot may be due to small sample effects or other bias factors, rather than substantial publication bias (Supplementary Figure 6).

### 3.7 Sensitivity analysis

The sensitivity analysis demonstrated that the impact of TCE on the QoL of Parkinson’s disease patients was relatively stable within the current dataset. By sequentially removing each included study and recalculating the overall effect size and confidence intervals, the results showed that regardless of which study was excluded, the effect size remained significant with only minor variations. This indicates that traditional Chinese exercises can significantly improve patients’ QoL. The sensitivity analysis suggests that bias or unique data from any single study does not substantially affect the overall conclusion, thereby enhancing the credibility and applicability of the research findings. This indicates that traditional Chinese exercises, as an intervention, have a reliable scientific basis for improving the QoL in Parkinson’s disease patients (Supplementary Figure 7).

## 4 Discussion

### 4.1 Main findings

**Figure 5:**
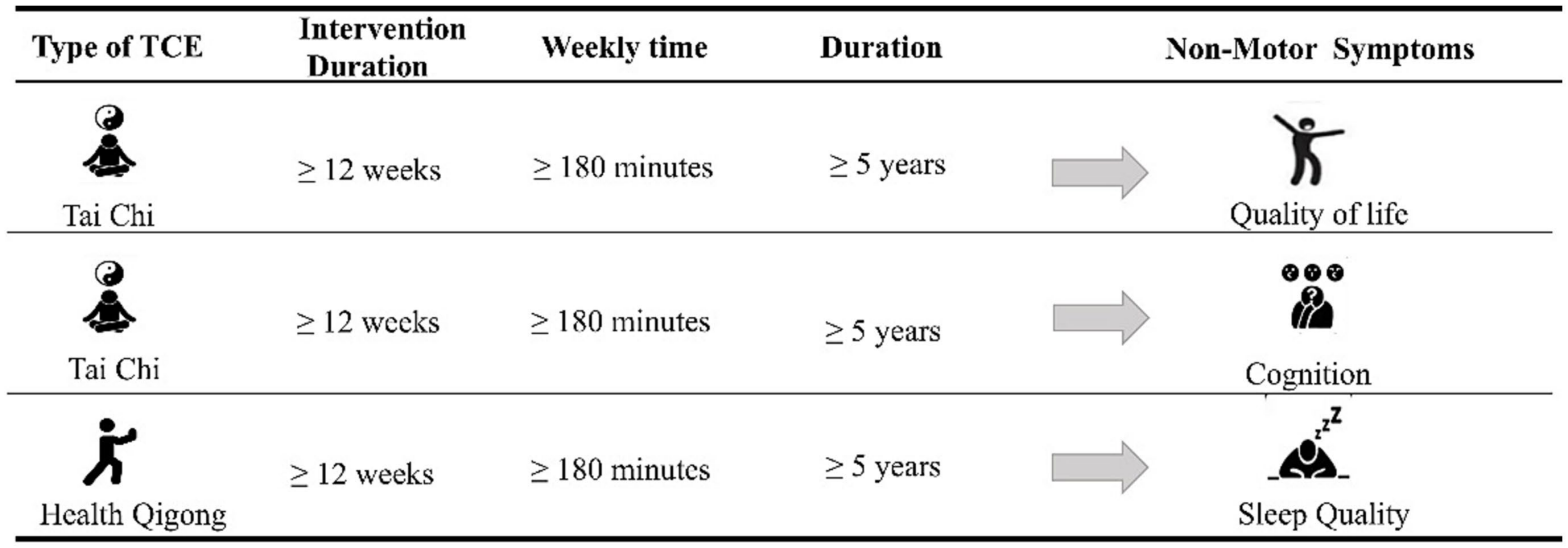
Main findings of traditional Chinese exercises on quality of life, cognition, sleep in PD.

This systematic review and meta-analysis suggests that TCE may improve cognition, sleep quality, and QoL in patients with PD. Crucially, through detailed subgroup analyses, we offer preliminary comparative insights into the differential efficacy of Tai Chi versus Health Qigong for specific NMS domains and propose a symptom-targeted “exercise prescription” framework based on intervention parameters (type, duration, frequency, weekly dose) and disease duration. The results indicated that, compared with Qigong, Tai Chi was more effective in improving cognition and QoL. Conversely, Health Qigong was more effective in improving sleep. Regarding intervention duration, ≥12 weeks demonstrated significant improvements across all outcomes (cognition, QoL, and sleep). For QoL specifically, both shorter (<12 weeks) and longer (≥12 weeks) durations were effective, though the longer duration showed a stronger effect size. High weekly doses (≥180 min) were consistently effective across all outcomes. This study also found that TCE was more effective for patients with a disease duration of ≥ 5 years (Figure 5).

### 4.2 Effects of traditional Chinese exercises in PD patients

Treating cognitive decline, sleep disturbances, and impaired quality of life in PD patients remains a clinical challenge. This difficulty is largely due to an incomplete understanding of the disease’s mechanisms and significant inter-patient heterogeneity. While the precise mechanisms for PD-related cognitive decline and sleep disturbances are not fully understood, research suggests several potential contributors: (1) Alterations in brain structure, including atrophy in the frontal, parietal, and temporal lobes and the hippocampus, as well as compromised white matter integrity in key tracts (e.g., the corpus callosum and superior longitudinal fasciculus). These structural changes consequently impair higher cognitive abilities, such as executive function, attention, and memory (Zhou et al., 2020; Niu et al., 2025). (2) Disturbances in neurotransmitter signaling, involving widespread dysfunction in the dopaminergic, cholinergic, noradrenergic, and serotonergic systems. These systems are critically linked to both cognitive decline and sleep regulation (Frouni et al., 2022; Samizadeh et al., 2023). (3) Neuroinflammation and decreased levels of brain-derived neurotrophic factor (BDNF). Reduced BDNF, in particular, is significantly associated with cognitive impairment and sleep disorders (e.g., REM sleep behavior disorder), leading to diminished neuroplasticity and synaptic dysfunction (Wang Y. et al., 2022; Zhao et al., 2025). (4) Circadian rhythm disturbances and hypothalamic dysfunction that disrupt the sleep–wake cycle. These issues can interfere with melatonin secretion, resulting in difficulty initiating sleep, nocturnal awakenings, and excessive daytime sleepiness (Videnovic and Golombek, 2013).

TCE, a unique form of mind–body exercise, have recently been shown to improve cognition, sleep, and QoL in PD patients through several potential mechanisms. From the standpoint of TCM theory, Tai Chi emphasizes the ‘unity of form and spirit’ and ‘guiding ‘Qi’ with the mind’; its complex training involving cognitive-motor integration may therefore be more conducive to ‘regulating the spirit’ and ‘enhancing cognitive function.’ In contrast, Health Qigong places greater emphasis on respiratory techniques and ‘regulating the breath,’ which may be more beneficial for ‘calming the spirit’ and improving sleep architecture. For cognition, TCE may promote neuroplasticity and neurotrophic factor regulation. Research indicates that regular Tai Chi training can significantly increase brain-derived neurotrophic factor (BDNF) levels, enhancing synaptic plasticity and neurodegenerative capacity (Gan et al., 2025). Tai Chi, an exercise integrating attentional demands and dual-task characteristics, requires practitioners to sustain attention, engage in procedural learning, and maintain spatial awareness. Consequently, one study suggested that the dual-task nature of Tai Chi may positively impact attentional performance in PD patients during working memory tasks (Taylor-Piliae et al., 2010). Functional magnetic resonance imaging (fMRI) studies have shown that long-term Tai Chi practice can improve functional connectivity (FC) within the Default Mode Network (DMN) and Frontoparietal Network (FPN) in PD patients, thereby enhancing memory function (Jasim et al., 2023). Second, TCE may inhibit neuroinflammation and improve sleep quality. Qigong has been shown to effectively reduce levels of pro-inflammatory cytokines, such as TNF-*α* and IL-1β (Moon et al., 2024). Notably, a reduction in TNF-α levels correlates significantly with improved Parkinson’s Disease Sleep Scale (PDSS) scores (Moon et al., 2017). Third, TCE may restore autonomic nervous system (ANS) balance. The deep breathing and meditation in TCE are thought to act as non-invasive vagus nerve stimulation, which enhances parasympathetic tone and improves heart rate variability (HRV; Larkey et al., 2024; Duan et al., 2025). This autonomic re-regulation is believed to reduce nocturnal hyperarousal and anxiety, thereby improving sleep architecture and quality. The observed differential efficacy between Tai Chi and Health Qigong might stem from inherent characteristics: Tai Chi’s greater cognitive-motor integration demands may preferentially enhance cognition and overall well-being, while Health Qigong’s stronger focus on breath regulation and deep relaxation could be particularly conducive to improving sleep architecture. These effects collectively contribute to the overall improvement in QoL for PD patients. Taken together, the available evidence suggests potential benefits of TCEs for non-motor symptoms in PD. Therefore, subgroup analyses were conducted to explore possible patterns of differential response across intervention characteristics.

### 4.3 Compared to related studies

Up to 40% of PD patients have mild cognitive impairment, and without further intervention, 23–40% of patients may progress to PD dementia (Janvin et al., 2006; Cong et al., 2022). Common outcome measures for assessing cognitive function in PD include the Montreal Cognitive Assessment (MoCA, *n* = 3) and the Mini-Mental State Examination (MMSE, *n* = 2). Our study provides preliminary symptom-specific insights based on subgroup analyses. In this study, TCE was found to exert a statistically significant effect on improving cognitive function. Tai Chi was found to be more effective than Health Qigong in enhancing cognitive performance, and the optimal effect was achieved with low frequency, moderate intensity (< 3 times per week and ≥180 min per week), and medium-term intervention (≥12 weeks). This finding is consistent with Peng’s study, which showed that high-dose (>200 min per week) and medium-term (13–24 weeks) TCE, such as Tai Chi, compared to Health Qigong, significantly improved MoCA scores (Peng et al., 2024). According to this study’s subgroup analysis, moderate-dose Tai Chi training would be more beneficial for enhancing cognitive function. This may stem from the discipline’s demanding multitasking requirements presenting a more effective challenge to the brain’s cognitive resources. The low-frequency schedule avoids cognitive fatigue, thereby optimizing the neuroplasticity-inducing effects. Alternatively, it may be due to its ability to improve adherence and reduce fatigue, which makes long-term participation easier. Tai Chi also includes training in mind–body coordination, dual-task processing, and focus, which are thought to be especially useful in avoiding cognitive impairment in PD (Janvin et al., 2006; Cong et al., 2022). It is crucial to remember, though, that longer weekly exercise sessions may have a negative impact on cognitive-related results rather than improving them. In contrast, our findings contradict those of Wang, who reported that 60–200 min per week for 6–10 weeks, or 60–120 min per week of mind–body exercises (including TCE, yoga, and dancing), improved cognitive function in older adults (Wang et al., 2021). The difference might result from a more expansive definition of mind–body exercise that includes not just TCE but also exercises like yoga and dancing. The diversity of study populations and cognitive testing instruments may also be to blame. This difference is further influenced by the small number of experiments that are exclusively focused on TCE. The necessity for future studies to focus on standardized TCE therapies to improve cognitive function in Parkinson’s disease is highlighted by this disparity.

Sleep problems are very common in people with PD. They affect between 60 to 98% of individuals, which can be broadly categorized into two main types: dyssomnias (including insomnia and hypersomnia) and parasomnias (Minakawa, 2022). These sleep disturbances may arise directly from PD-related neurodegeneration, or be affected by comorbidities (e.g., depression, anxiety) and side effects of pharmacotherapy. TCE has been shown to help PD patients with their sleep issues (Chen et al., 2020; Peng et al., 2024). Based on the PD Sleep Scale-2 (PDSS-2) and PDSS scores, our subgroup analyses suggested that Health Qigong practiced for ≥12 weeks and ≥180 min per week may be associated with more favorable sleep outcomes. These results agree with the World Health Organization’s suggested recommendations for the frequency, duration, and dosage of exercise programs (Bull et al., 2020). Moreover, high-frequency health qigong may prove more beneficial for sleep precisely because it emphasizes respiratory regulation and relaxation. Such intensive practice may effectively restore healthy sleep architecture by continuously modulating circadian rhythms and reducing sympathetic nervous system activity. However, clinical trials investigating the use of TCE for treating sleep disorders in PD remain relatively limited, and there is a lack of standardized, analyzable outcome indicators. Further well-designed studies are needed to elucidate the efficacy of TCE in improving sleep-related symptoms in this population.

QoL in patients with PD is commonly assessed using instruments such as the PDQ, PDQ-39, PDQ-8, and PD-QoL (Peto et al., 1995; de Boer et al., 1996; Kumar et al., 2020). Previous meta-analyses have shown that traditional mind–body practices such as Tai Chi and Qigong significantly improve the QoL of PD patients when the intervention duration is at least 12 weeks (Song et al., 2017; Chen et al., 2020). Furthermore, there is evidence that longer-term RCTs (>6 months, with the longest follow-up at 9 months) can further significantly improve QoL (Wan et al., 2021). Our findings are generally consistent with those of previous studies. Peng et al. reported that moderate-duration (13–24 weeks) Eastern exercise interventions are more effective in improving QoL in PD patients (Peng et al., 2024). In addition to consolidating short-term advantages, consistent, long-term periodic exercise may slow the onset of non-motor symptoms by preserving neuroplasticity, improving autonomic nervous system regulation, and lowering levels of chronic inflammation (Song et al., 2017; Chen et al., 2020). Therefore, in order to obtain long-lasting benefits in QoL, more focus should be placed on long-term adherence and the development of exercise routines.

This study also found that TCE was most effective in improving cognition, SD and QoL in PD patients with a disease duration of ≥ 5 years. Currently, there is limited research on the relationship between NMS and disease progression in PD. Existing studies indicate that cognitive decline, and sleep disturbances in PD patients are variable yet persistent, often worsening with disease progression and reducing the likelihood of symptom relief (Daniels et al., 2024). These findings may be explained by the different pathophysiological mechanisms and subjective symptom presentations at various stages of the disease. As PD progresses, structural and functional deterioration in brain regions such as the prefrontal-striatal pathway and limbic system becomes more pronounced, resulting in more severe cognitive and emotional impairments. Patients with mild to moderate PD and a longer disease duration may exhibit stronger subjective engagement with interventions, leading to more significant improvements (Petzinger et al., 2013; Mak et al., 2017). Conversely, patients with shorter disease duration often experience prominent sleep disturbances, which are among the earliest NMS of PD (Chahine et al., 2017; Ivy et al., 2021). As a mind–body integrative exercise, TCE can regulate the autonomic nervous system and reduce physical tension, thereby improving sleep quality in early-stage patients. Furthermore, among the included studies, 4 focused on patients with disease duration <5 years, and 9 on those with duration ≥5 years. This suggests an insufficient number of studies focusing on patients with shorter disease duration.

### 4.4 Limitations

This study has several limitations. First, the literature search was restricted to English-language publications, and most of the included studies were conducted in China, which may limit the generalizability of the findings and potential publication bias. Second, some of the included studies had small sample sizes, which may reduce the statistical power and stability of the results. Most included studies were pilot or early-phase randomized controlled trials, which limits the maturity of the evidence base and the certainty of the conclusions. Third, the control groups across studies employed varying intervention methods, which could have introduced confounding effects on the outcomes. Lastly, several subgroup analyses were based on a limited number of studies and should therefore be regarded as exploratory and hypothesis-generating rather than confirmatory. Although patients were staged based on disease duration, the clinical stage of PD was not considered, which may have introduced potential bias into the results.

Although an increasing body of evidence suggests that TCE may provide potential benefits for improving cognitive function, sleep quality, depressive symptoms, and overall QoL in patients with PD, the underlying mechanisms remain poorly understood, and the current evidence base is still incomplete. Future research should prioritize large-scale, methodologically rigorous studies to elucidate the precise therapeutic mechanisms of TCE in PD. In the future, it will be necessary to tailor exercise interventions by customizing the type, dosage, and duration according to an individual’s specific s NMS and the severity of their disease. At present, most evaluations of TCE effectiveness rely on subjective outcome measures such as rating scales; thus, incorporating more objective and quantitative assessment tools in future studies will be essential. Moreover, there is a need to further standardize clinical protocols involving TCE for PD to optimize rehabilitation strategies and maximize patient outcomes.

## 5 Conclusion

This meta-analysis provides the current evidence suggests that TCE encompassing Tai Chi and Health Qigong, as a beneficial intervention for cognitive function, sleep quality, and QoL in Parkinson’s disease. Our novel findings through subgroup analysis provide preliminary insights into symptom-specific patterns of intervention effects(1)For cognitive enhancement: May be prioritized Tai Chi, delivered at a moderate-to-high total weekly exercise volume (≥180 min), over ≥12 weeks.(2)For improving sleep disturbances: May be prioritized Health Qigong, delivered at a weekly volume (≥180 min), over ≥12 weeks.(3)For enhancing QoL: Tai Chi appears superior, with interventions ≥12 weeks and ≥180 min weekly volume recommended.(4)Patients with longer disease duration (≥5 years) may derive greater benefits, particularly for cognition and QoL. Nonetheless, the current evidence remains limited, and further research is warranted. Future studies should employ stratified designs to determine the optimal type, dosage, and duration of TCE tailored to specific clinical phenotypes, neuroimaging techniques and biomarkers should be utilized to elucidate the neurobiological mechanisms by which TCE ameliorates non-motor symptoms of PD, efforts should be directed toward developing objective assessment tools to mitigate the bias inherent in subjective scales thereby enabling more personalized and effective rehabilitation strategies for individuals with PD.

## Data Availability

The original contributions presented in the study are included in the article/Supplementary material, further inquiries can be directed to the corresponding authors.
