# Supplementary material for: Traditional Chinese exercise for quality of life, cognition, sleep in Parkinson’s disease: a systematic review and meta-analysis
Source: Front Psychol. 2026 Jun 9;17:1824910. doi: 10.3389/fpsyg.2026.1824910 (PMC13286797; doi:10.3389/fpsyg.2026.1824910)

**Supplementary Material**

**Traditional Chinese Exercises for Quality of Life, Cognition, Sleep in Parkinson’s Disease: A Systematic Review and Meta-Analysis**

Table S1. Search strategy ............................................................................................. 2

Table S2. Literature quality assessment ........................................................................ 5

Figure S1. Literature quality assessment ....................................................................... 6

Figure S2. QoL Subgroup analysis ................................................................................ 7

Figure S3. COG Subgroup analysis ............................................................................... 9

Figure S4. SQ Subgroup analysis ................................................................................... 11

Figure S5. Meta-regression bubble plots ........................................................................ 13

Figure S6. Publication bias Funnel plot ........................................................................14

Figure S7. Sensitivity analysis results ........................................................................15

**TABLE 1**

| **Pubmed** | ((((("Tai Ji"[Mesh]) OR ((((((((((Tai-ji[Title/Abstract]) OR (Tai Chi[Title/Abstract])) OR (Chi, Tai[Title/Abstract])) OR (Tai Chi Chuan[Title/Abstract])) OR (Taiji[Title/Abstract])) OR (Taijiquan[Title/Abstract])) OR (T'ai Chi[Title/Abstract])) OR (Tai Ji Quan[Title/Abstract])) OR (Ji Quan, Tai[Title/Abstract])) OR (Quan, Tai Ji[Title/Abstract]))) OR (("Qigong"[Mesh]) OR ((Ch'i Kung[Title/Abstract]) OR (Qi Gong[Title/Abstract])))) OR (((((baduanjin[Title/Abstract]) OR (ba duan jin[Title/Abstract])) OR (wuqinxi[Title/Abstract])) OR ((yijinjing[Title/Abstract]) OR (yi jinjing[Title/Abstract]))) OR (((((Chinese traditional exercise[Title/Abstract]) OR (traditional exercise[Title/Abstract])) OR (traditional Chinese exercise[Title/Abstract])) OR (Chinese exercise[Title/Abstract])) OR (mind-body exercise[Title/Abstract])))) AND (("Parkinson Disease"[Mesh]) OR ((((((((((Lewy Body Parkinson's Disease[Title/Abstract]) OR (Parkinson's Disease, Idiopathic[Title/Abstract])) OR (Parkinson's Disease, Lewy Body[Title/Abstract])) OR (Paralysis Agitans[Title/Abstract])) OR (Parkinson's Disease[Title/Abstract])) OR (Idiopathic Parkinson Disease[Title/Abstract])) OR (Lewy Body Parkinson Disease[Title/Abstract])) OR (Primary Parkinsonism[Title/Abstract])) OR (Parkinsonism, Primary[Title/Abstract])) OR (Parkinson Disease, Idiopathic[Title/Abstract])))) AND (((randomized controlled trial[Title/Abstract]) OR (randomized[Title/Abstract])) OR (placebo[Title/Abstract])) |
| --- | --- |

| **Cochrane library** | (((Tai Ji)MeSH OR (Tai-Ji):ti,ab OR (Tai Chi):ti,ab OR (Chi Tai):ti,ab OR (Tai Ji Quan):ti,ab OR (Quan Tai Ji):ti,ab OR (Ji Quan Tai):ti,ab OR (Taiji):ti,ab OR (Taijiquan):ti,ab OR (T'ai Chi):ti,ab OR (Tai Chi Chuan):ti,ab OR (Qigong)MeSH OR (Qi Gong):ti,ab OR (Chi Kung):ti,ab OR (yijinjing):ti,ab OR (baduanjin):ti,ab OR (wuqinxi):ti,ab OR (traditional Chinese exercise):ti,ab) AND ((Parkinson's Disease):ab,ti OR (Idiopathic Parkinson's Disease):ab,ti OR (Lewy Body Parkinson's Disease):ab,ti OR (Parkinson's Disease, Idiopathic):ab,ti OR (Parkinson's Disease, Lewy Body):ab,ti OR (Parkinson Disease, Idiopathic):ab,ti OR (Idiopathic Parkinson Disease):ab,ti OR (Lewy Body Parkinson Disease):ab,ti OR (Primary Parkinsonism):ab,ti OR (Parkinsonism, Primary):ab,ti OR (Paralysis Agitans):ab,ti) AND ((randomized controlled trial):ti,ab, OR (clinical trial):ti,ab, OR (randomized):ti,ab OR (randomization):ti,ab)) |
| --- | --- |

| **Embase** | |
| --- | --- |
| #1 | (("Tai Ji":ab,ti OR "Tai-Ji":ab,ti OR "Tai Chi":ab,ti OR "Chi Tai":ab,ti OR "Tai Ji Quan":ab,ti OR "Quan Tai Ji":ab,ti OR "Ji Quan Tai":ab,ti OR "Taiji":ab,ti OR "Taijiquan":ab,ti OR "Tai Chi Chuan":ab,ti OR "Qigong":ab,ti OR "Qi Gong":ab,ti OR "Chi Kung":ab,ti OR "yijinjing":ab,ti OR "baduanjin":ab,ti OR "wuqinxi":ab,ti OR "traditional Chinese exercise":ab,ti) |
| #2 | ("Parkinson's Disease":ab,ti OR "Idiopathic Parkinson's Disease":ab,ti OR "Lewy Body Parkinson's Disease":ab,ti OR "Parkinson's Disease, Idiopathic":ab,ti OR "Parkinson's Disease, Lewy Body":ab,ti OR "Parkinson Disease, Idiopathic":ab,ti OR "Idiopathic Parkinson Disease":ab,ti OR "Lewy Body Parkinson Disease":ab,ti OR "Primary Parkinsonism":ab,ti OR "Parkinsonism, Primary":ab,ti OR "Paralysis Agitans":ab,ti) |
| #3 | ("randomized controlled trial":ab,ti OR "clinical trial" OR "randomization":ab,ti OR "randomized":ab,ti)) |
| #4 | #1 AND #2 AND #3 |

| **Web of Science** | ((((((((((TS=(Parkinson's Disease)) OR TS=(Idiopathic Parkinson's Disease)) OR TS=(Lewy Body Parkinson's Disease)) OR TS=(Parkinson's Disease, Idiopathic)) OR TS=(Parkinson's Disease, Lewy Body)) OR TS=(Parkinson's Disease, Idiopathic)) OR TS=(Idiopathic Parkinson Disease )) OR TS=(Lewy Body Parkinson Disease)) OR TS=(Primary Parkinsonism)) OR TS=(Parkinsonism, Primary)) OR TS=(Paralysis Agitans (((((((((((((((((TS=(Tai Ji)) OR TS=(Tai-ji)) OR TS=(Tai Chi)) OR TS=(Chi Tai)) OR TS=(Tai Ji Quan)) OR TS=(Quan Tai Ji)) OR TS=(Ji Quan Tai)) OR TS=(Taiji)) OR TS=(Taijiquan)) OR TS=(T'ai Chi )) OR TS=(Tai Chi Chuan)) OR TS=(Qigong)) OR TS=(Qi Gong)) OR TS=(Chi Kung)) OR TS=( yijinjing)) OR TS=( baduanjin )) OR TS=(wuqinxi)) OR TS=(traditional Chinese exercise)((TS=(randomized controlled trial)) OR TS=(randomized)) OR TS=(placebo) |
| --- | --- |

| EBSCO | |
| --- | --- |
| S1 | AB Tai-ji OR AB Tai Chi OR AB Chi, Tai OR AB Tai Chi Chuan OR AB Taiji OR AB Taijiquan OR AB T'ai Chi OR AB Tai Ji Quan OR AB Ji Quan, Tai OR AB Quan, Tai Ji OR AB Ch'i Kung OR AB Qi Gong OR AB yijinjing OR AB baduanjin OR AB traditional Chinese exercise OR AB wuqinxi OR |
| S2 | AB Parkinson Disease OR AB Lewy Body Parkinson's Disease OR AB Parkinson's Disease, Idiopathic OR AB Parkinson's Disease, Lewy Body OR AB Paralysis Agitans OR AB Parkinson's Disease OR AB Idiopathic Parkinson Disease OR AB Lewy Body Parkinson Disease OR AB Primary Parkinsonism OR AB Parkinsonism, Primary OR AB Parkinson Disease, Idiopathic OR |
| S3 | AB randomized controlled trial OR AB randomized OR AB placebo OR |
| S4 | S1 AND S2 AND S3 |

Detailed search terms for each literature database

**TABLE 2**

| Study year | Random sequence generation | Allocation concealment | blinding of outcome assessors | incomplete outcome | selective outcome reporting | other risks of bias |
| --- | --- | --- | --- | --- | --- | --- |
| Li 2024 | Low | low | low | High | low | low |
| ZHU 2020 | High | High | High | High | Unclear | High |
| Li 2022 | Low | Low | Low | Low | low | low |
| Yin 2025 | unclear | unclear | Low | Low | Low | Low |
| Li 2013 | low | Low | low | Low | Low | low |
| Moon 2020 | Low | Low | Low | High | low | low |
| Moon 2017 | Unclear | Unclear | Low | Low | Low | High |
| Nocera 2013 | High | High | High | High | Unclear | High |
| Vergara 2018 | Low | Low | Low | low | Low | Low |
| Wang 2022 | Low | Low | Low | Low | Low | Low |
| Xiao 2015 | Low | Unclear | Low | Low | Low | Low |
| Poier  2019 | Low | Unclear | Low | High | Low | Low |
| Cheon 2013 | High | High | Low | High | Low | High |
| Li 2023 | Low | Low | Low | Low | Low | High |
| Tsai 2025 | Unclear | Unclear | Low | Low | Low | High |

Quality assessment of included studies

**Figure 1.**


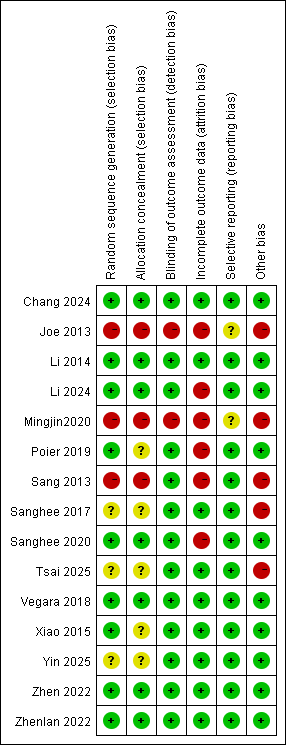

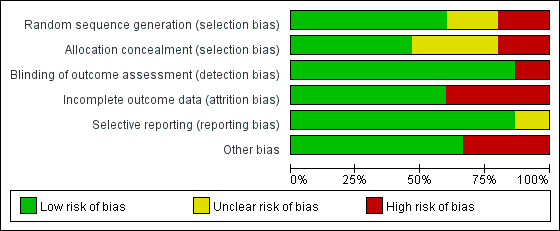


Risk of bias of included studies.

**Figure 2.**


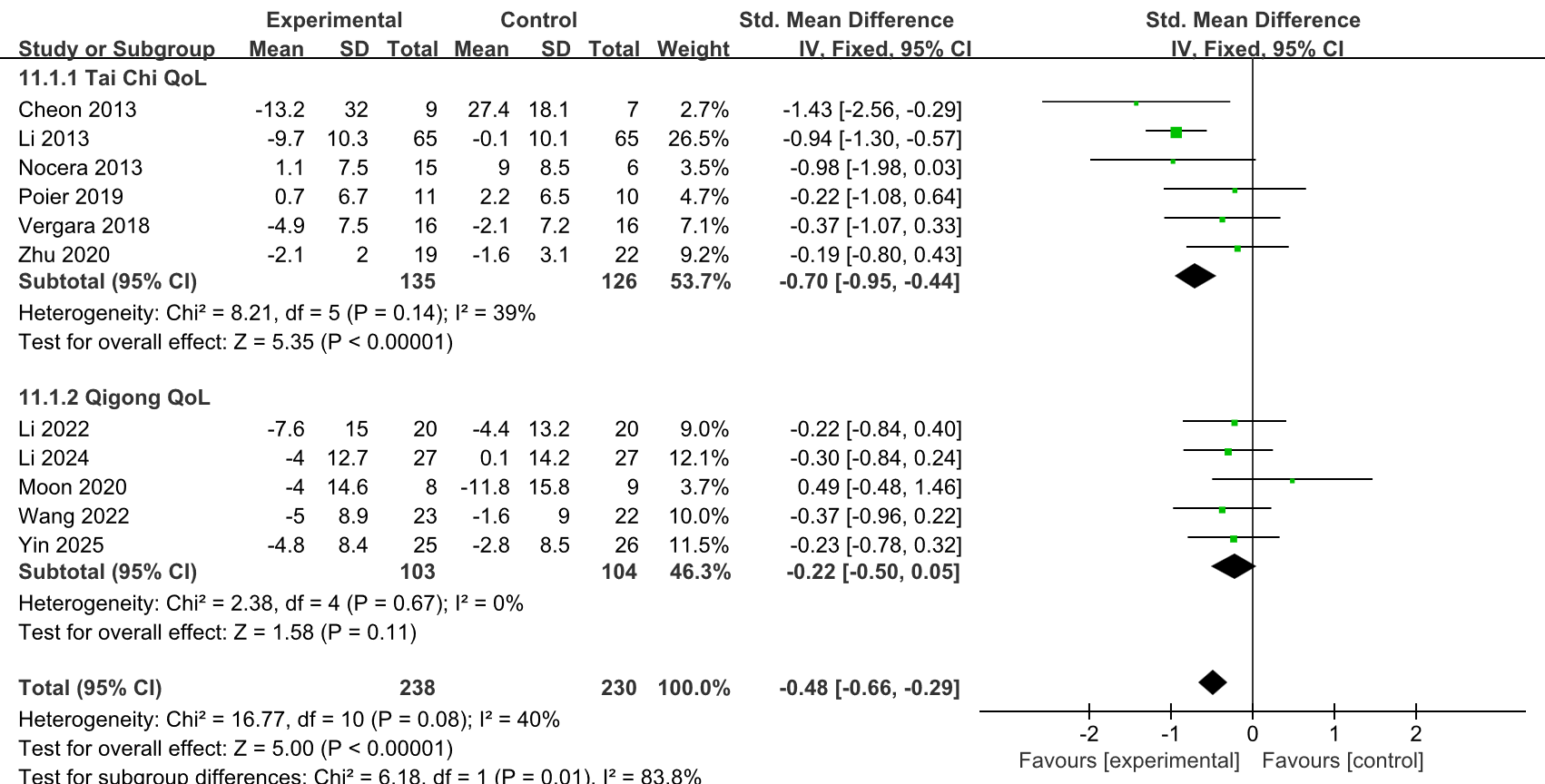


Subgroup analyses results of the effect of type of intervention on QoL in PD patients


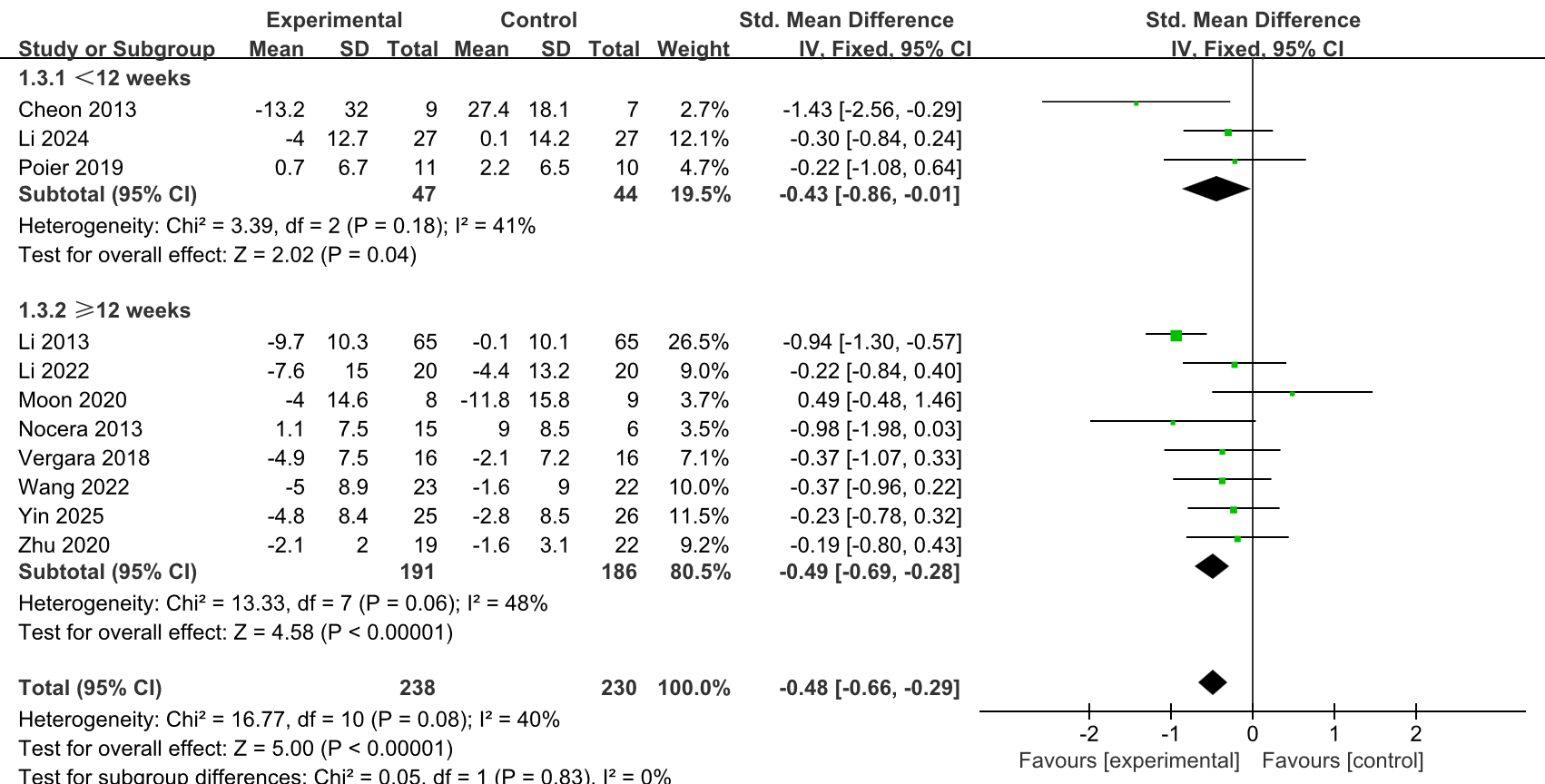


Subgroup analyses results of the effect of duration of intervention on QoL in PD patients


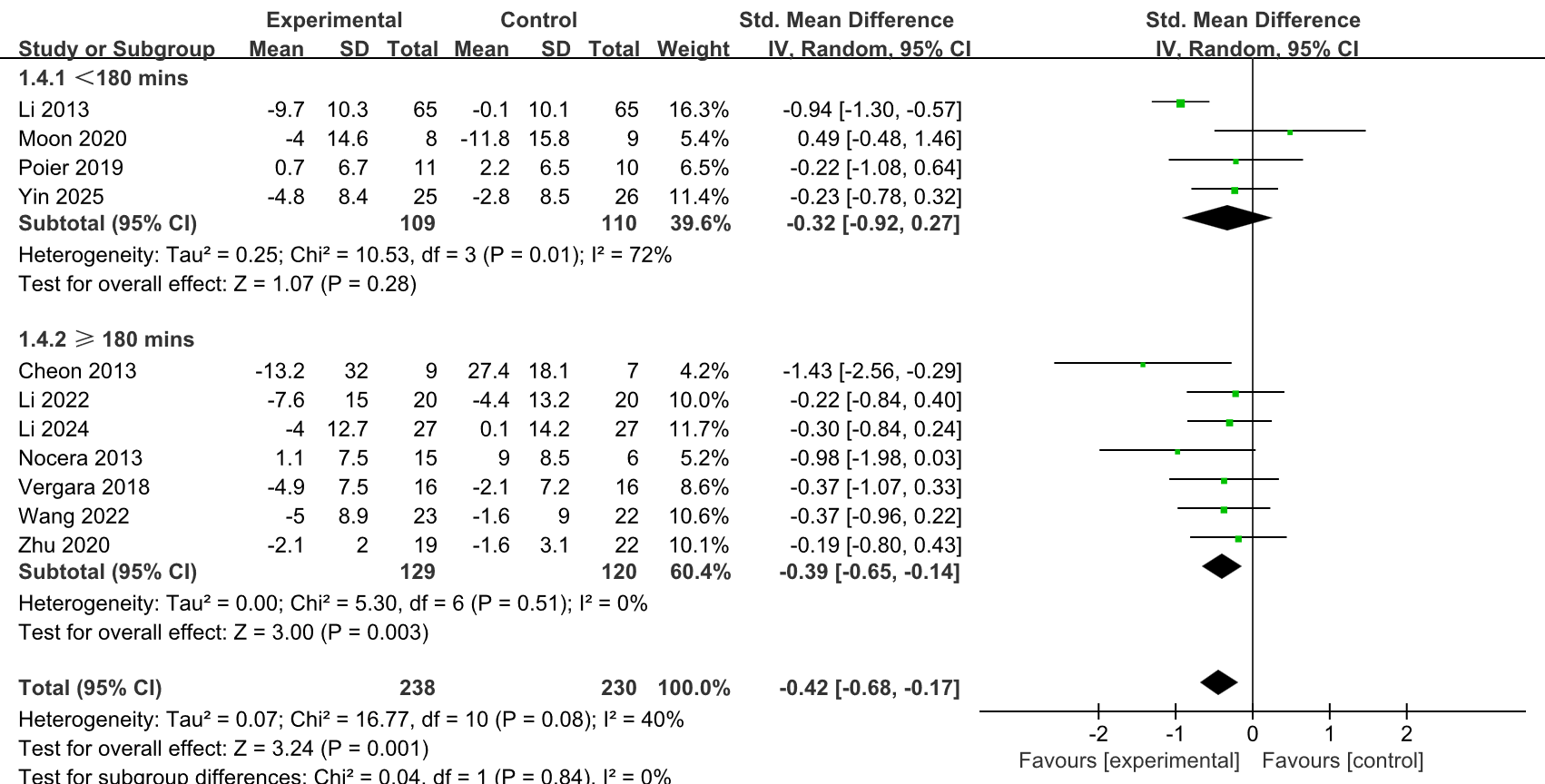


Subgroup analyses results of the effect of weekly time of intervention on QoL in PD patients


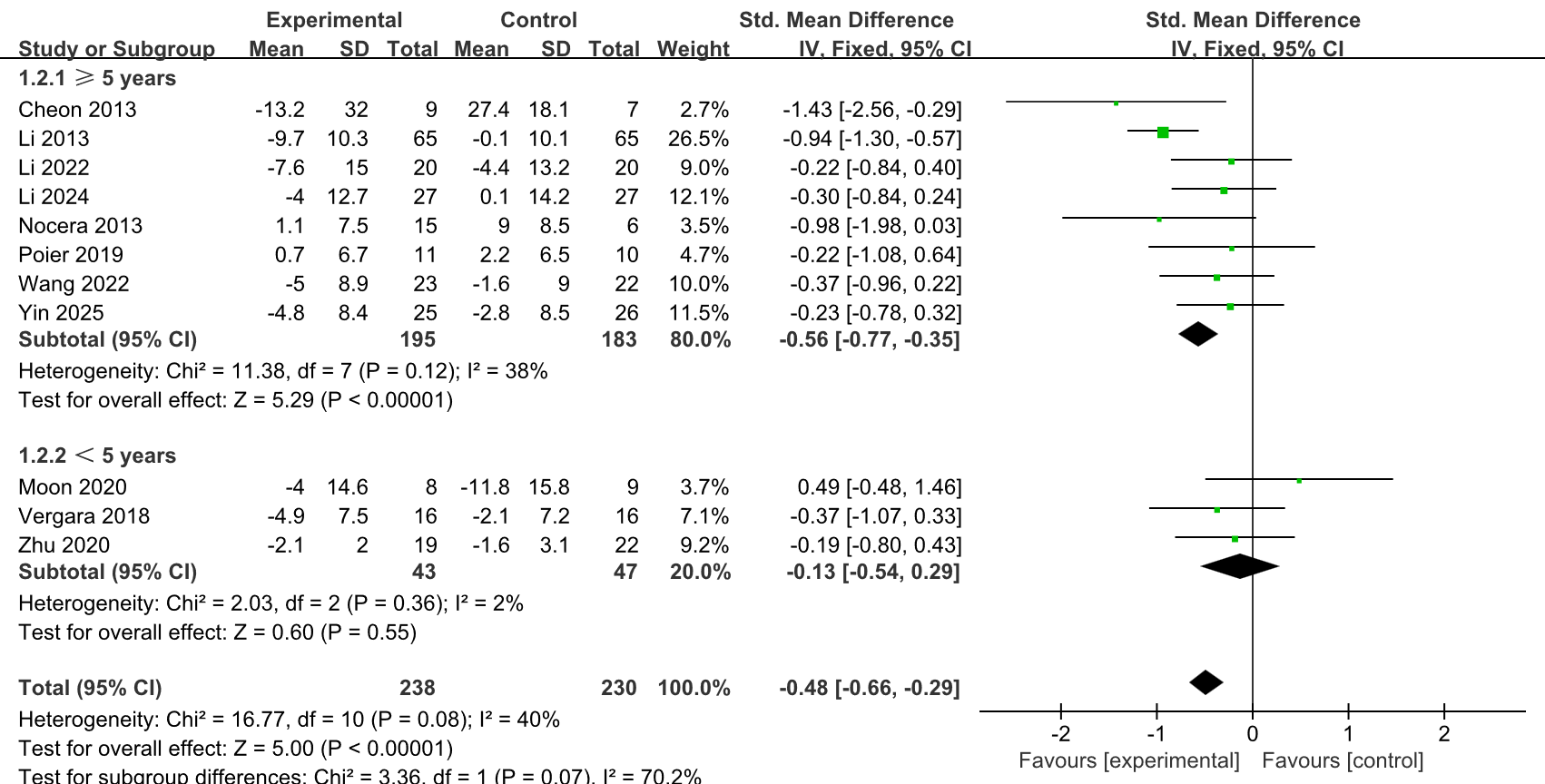


Subgroup analyses results of the effect of disease duration of intervention on QoL in PD patients

**Figure 3.**


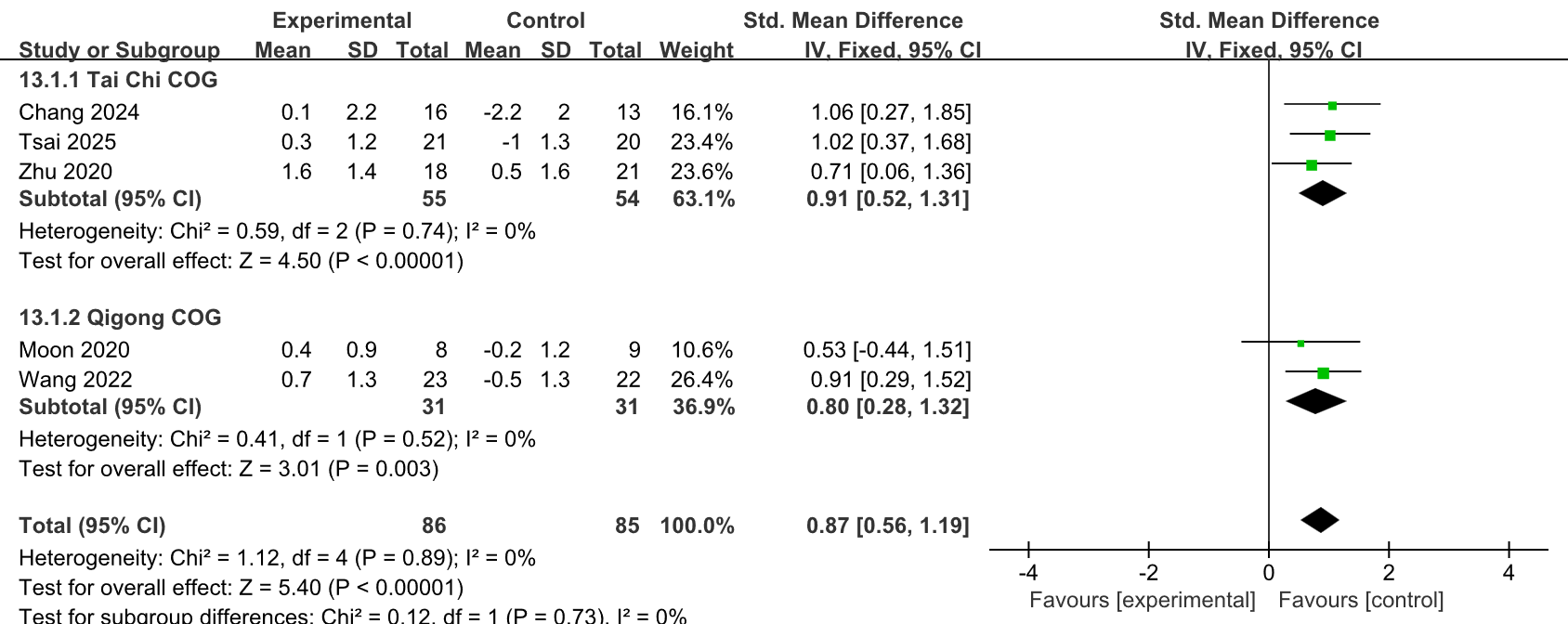


Subgroup analyses results of the effect of type of intervention on cognition in PD patients


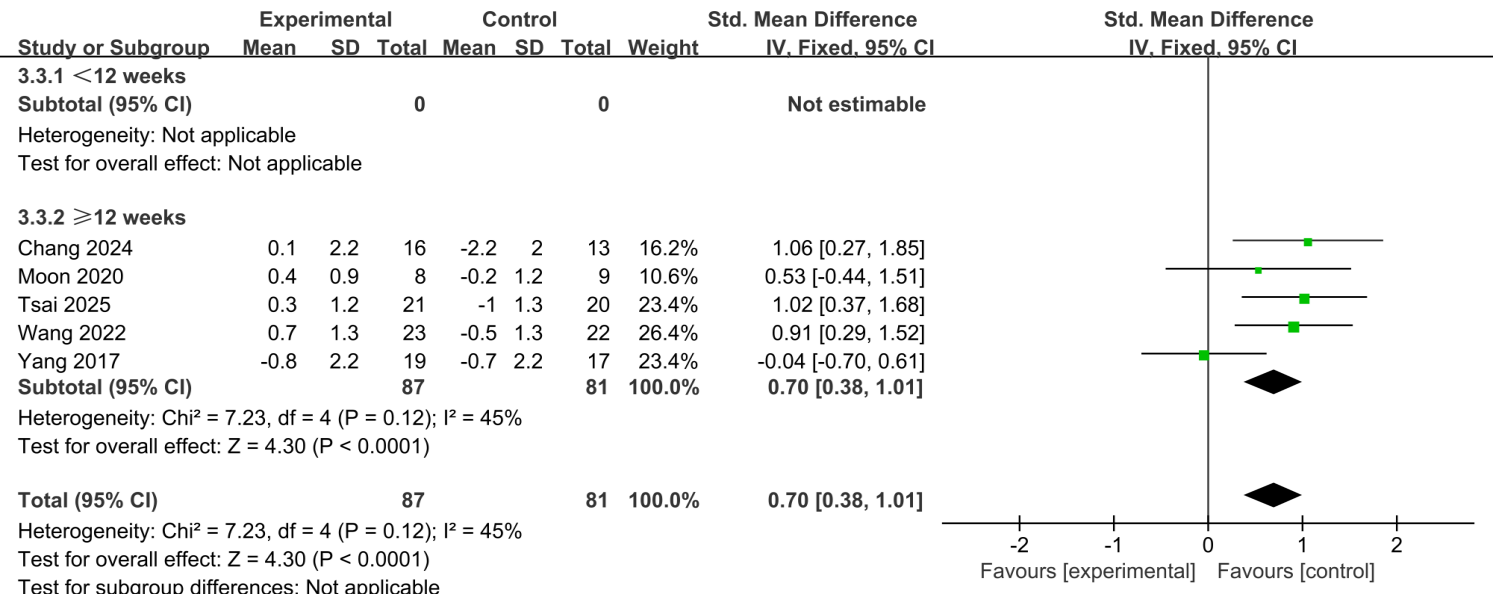
Subgroup analyses results of the effect of duration of intervention on cognition in PD patients


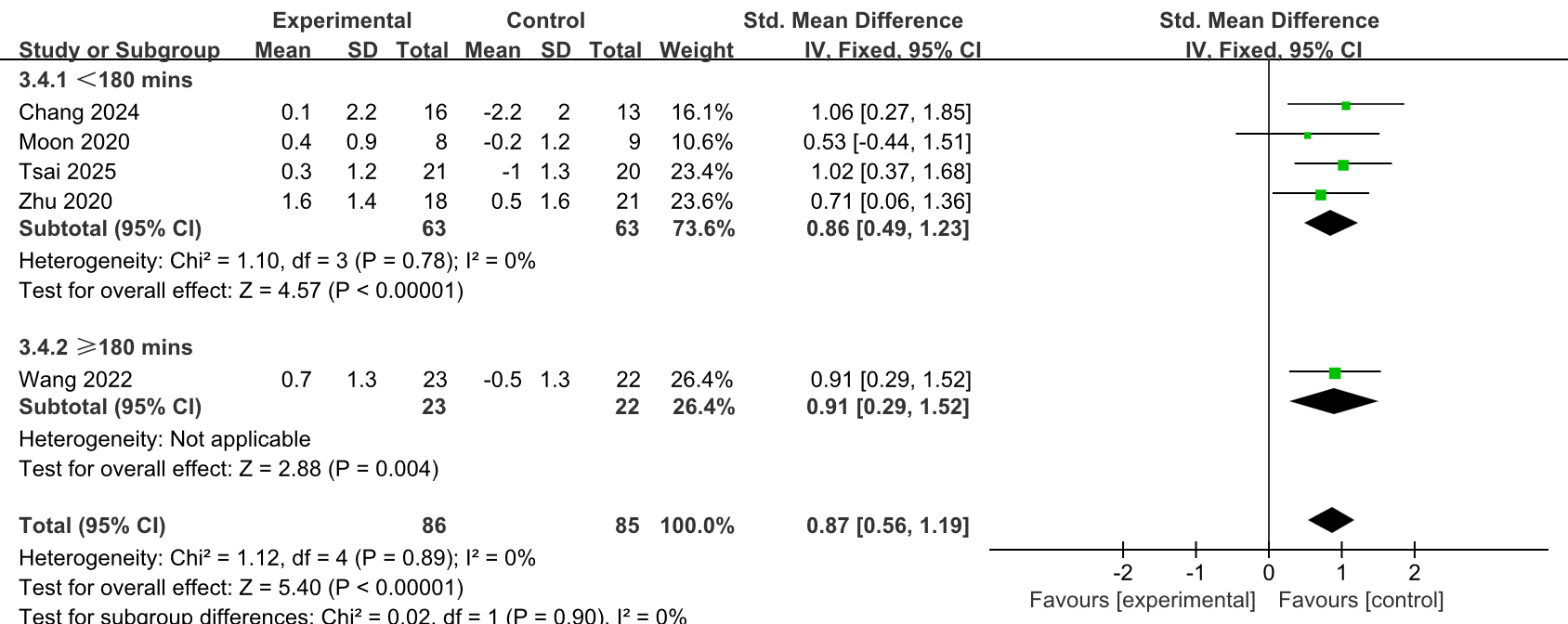


Subgroup analyses results of the effect of weekly time of intervention on cognition in PD patients


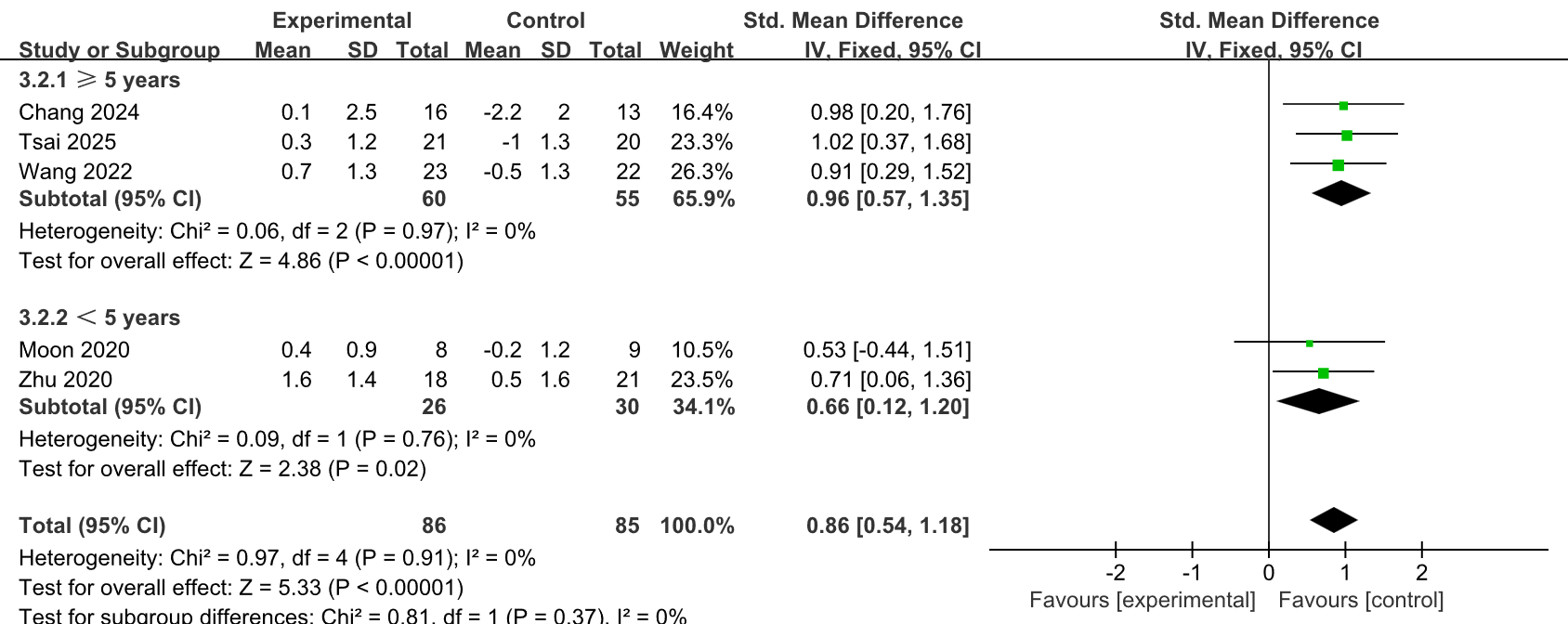


Subgroup analyses results of the effect of disease duration of intervention on cognition in PD patients

**Figure 4.**


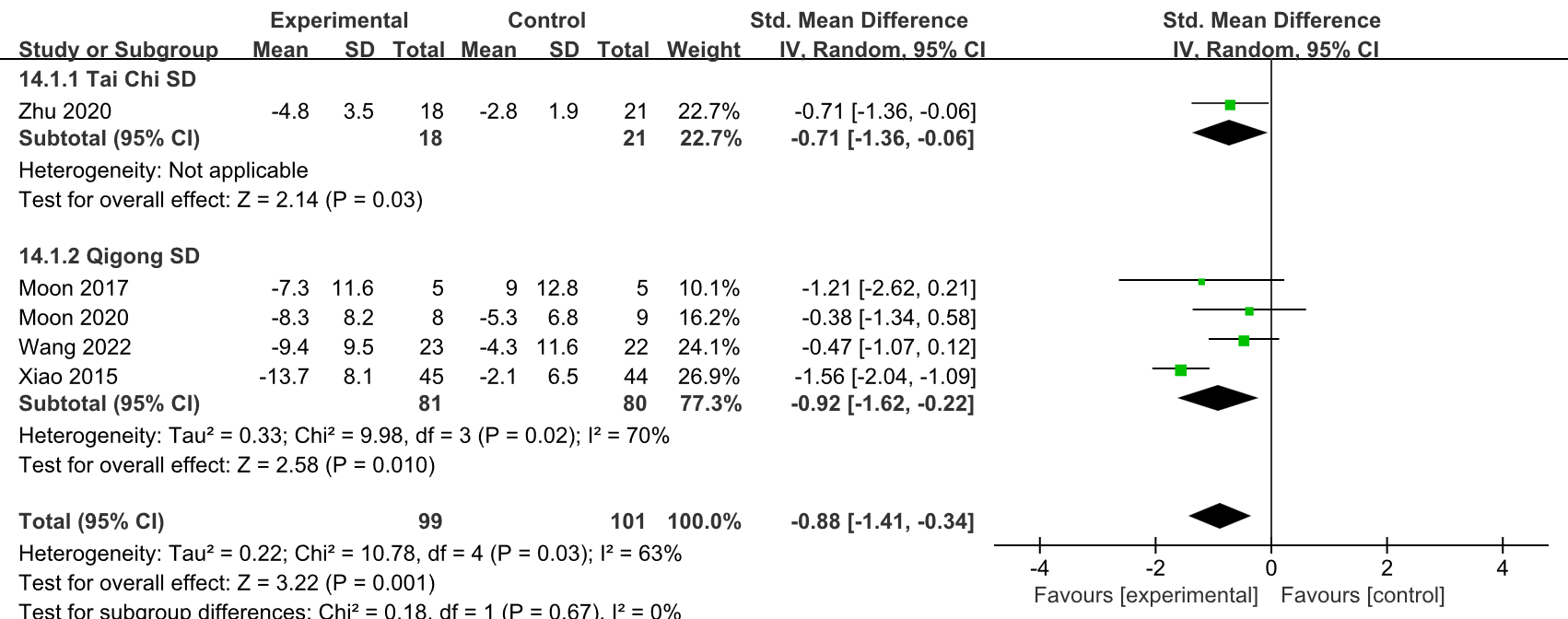


Subgroup analyses results of the effect of type of intervention on sleep quality in PD patients


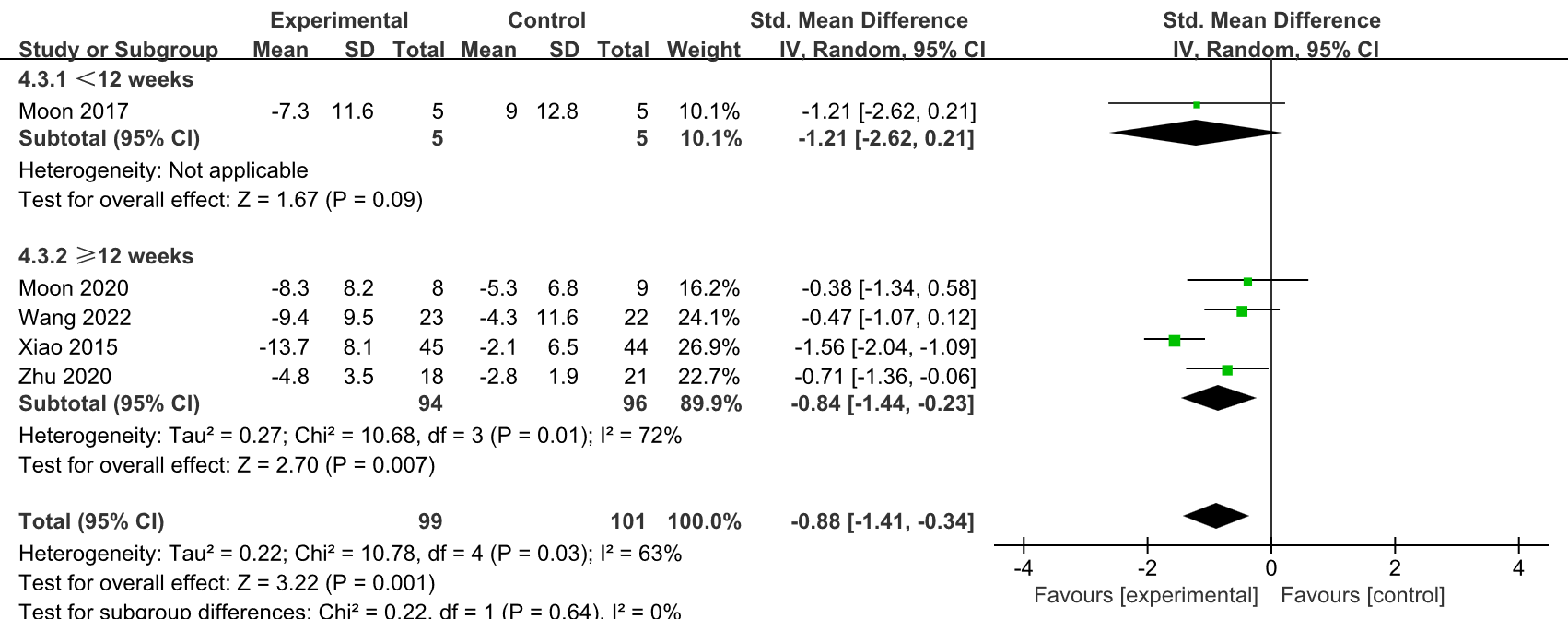


Subgroup analyses results of the effect of duration of intervention on sleep quality in PD patients


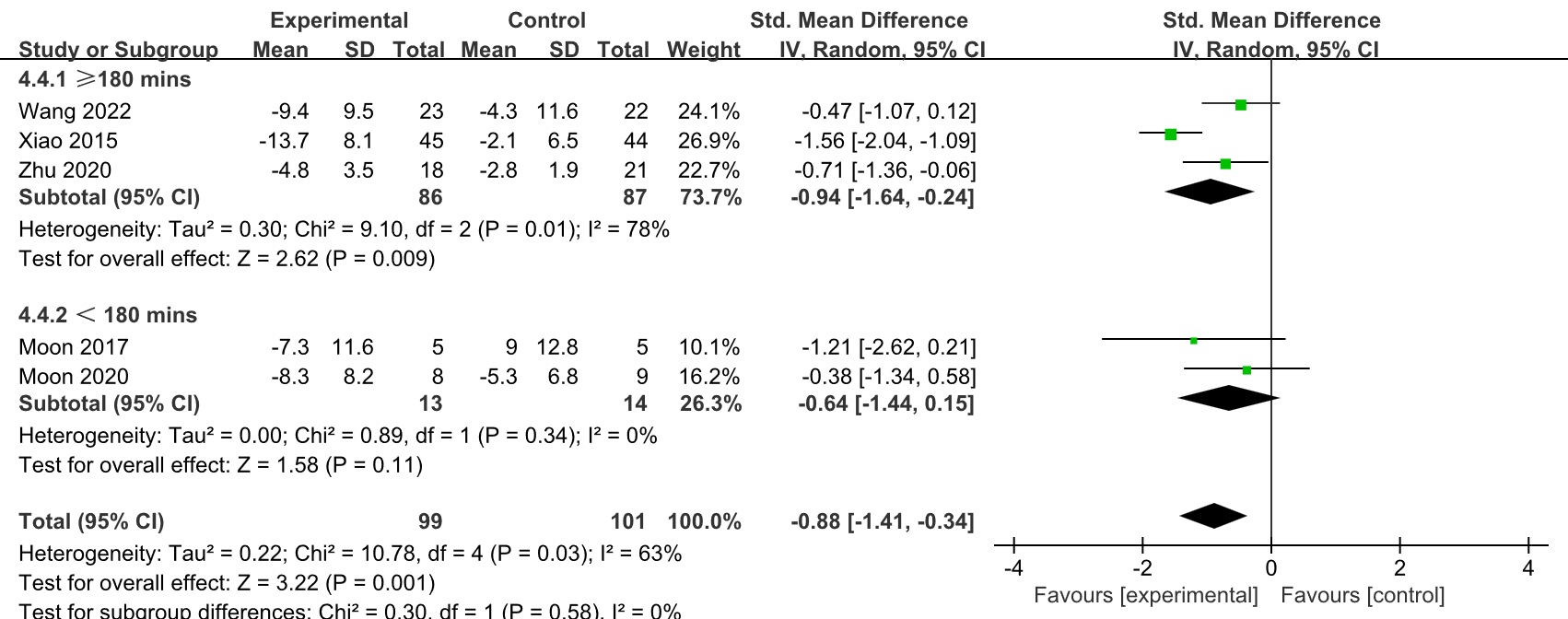


Subgroup analyses results of the effect of weekly time of intervention on sleep quality in PD patients


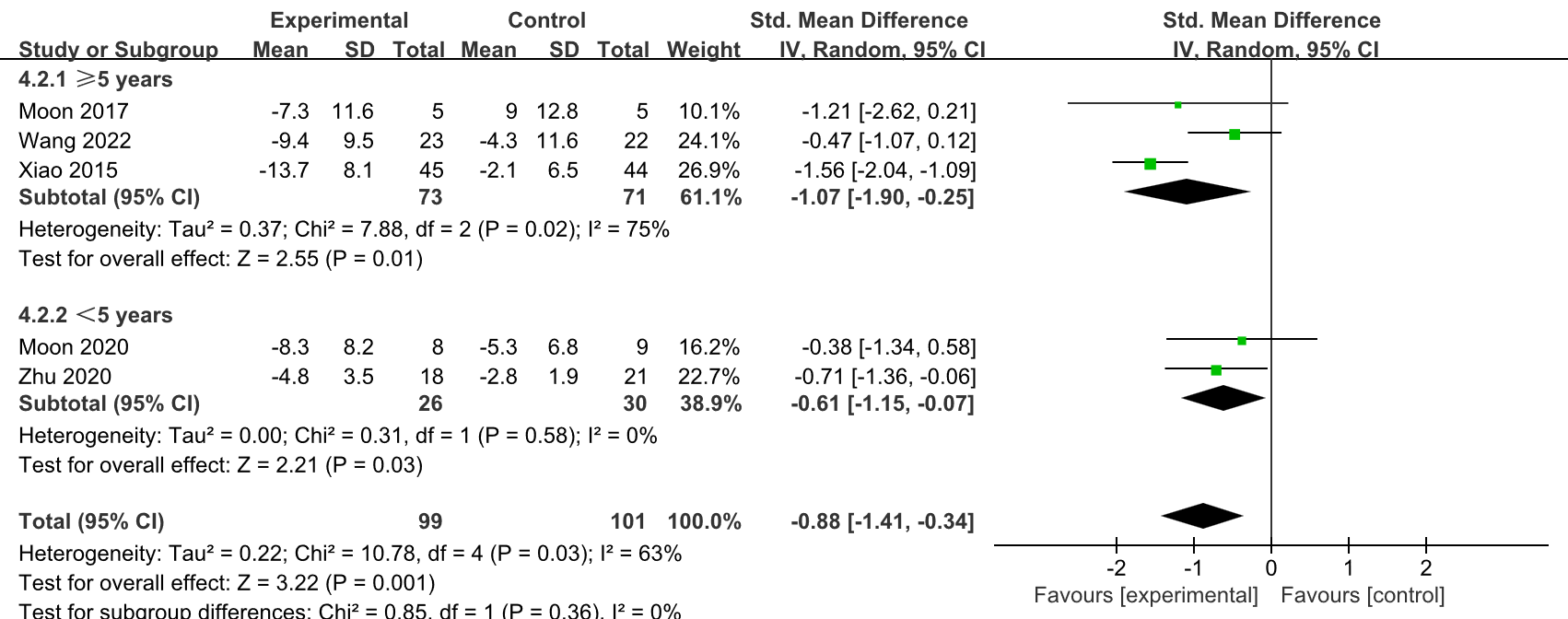


Subgroup analyses results of the effect of disease duration of intervention on sleep quality in PD patients

**Figure 5.**

| 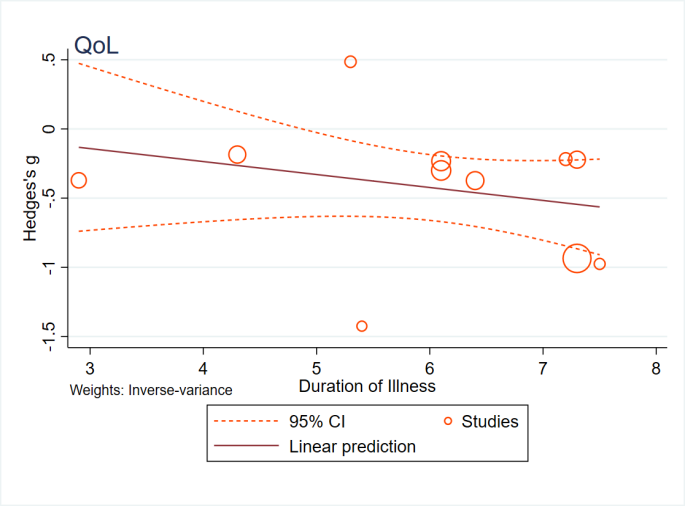 | 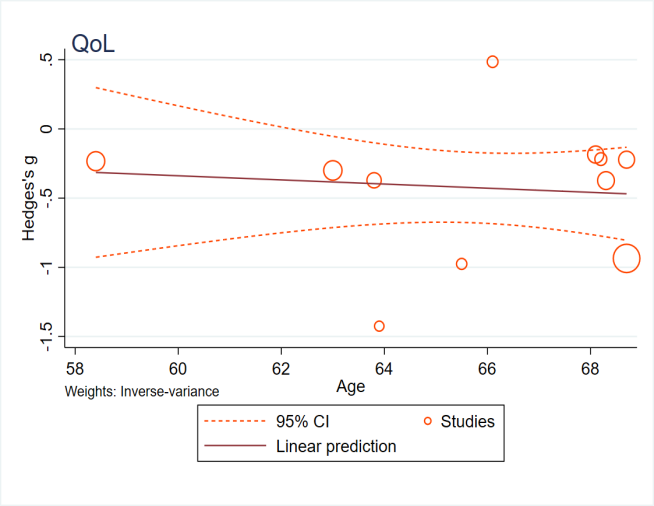 |
| --- | --- |
| (A) | (B) |
| 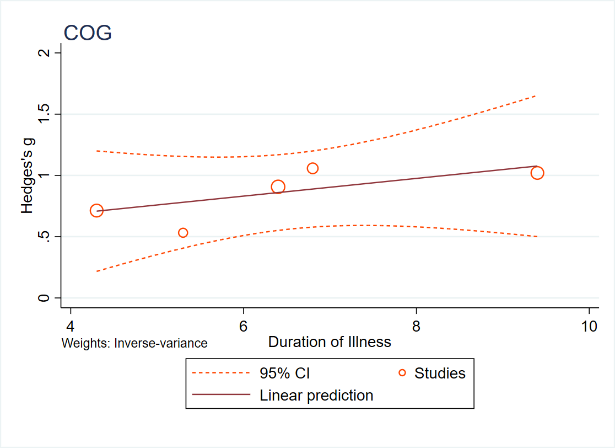 | 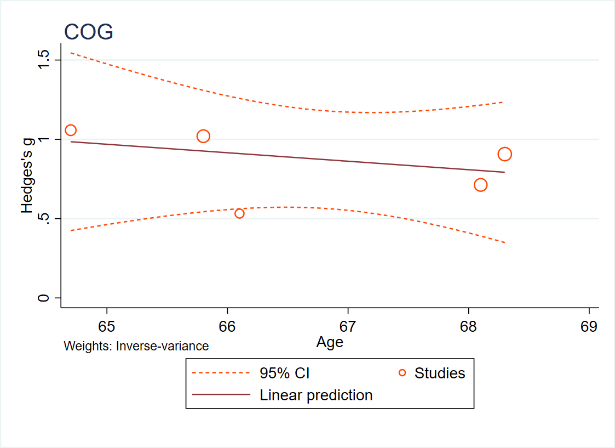 |
| (C) | (D) |
| 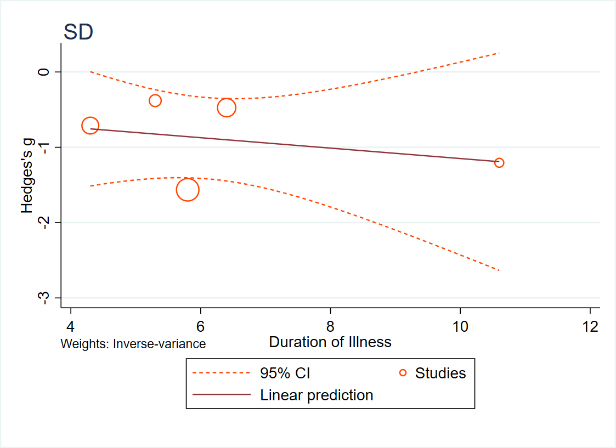 | 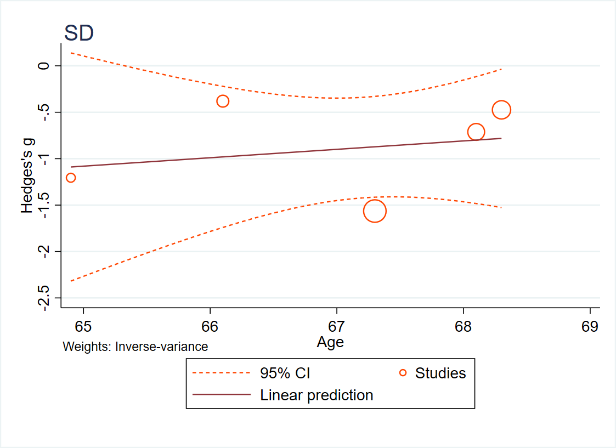 |
| (E) | (F) |
| Meta-regression bubble plots were conducted to examine the associations of differences in quality of life(A)(B), cognition (C)(D), and sleep disorders (E)(F) with duration of illness and age. | |

**Figure 6.**


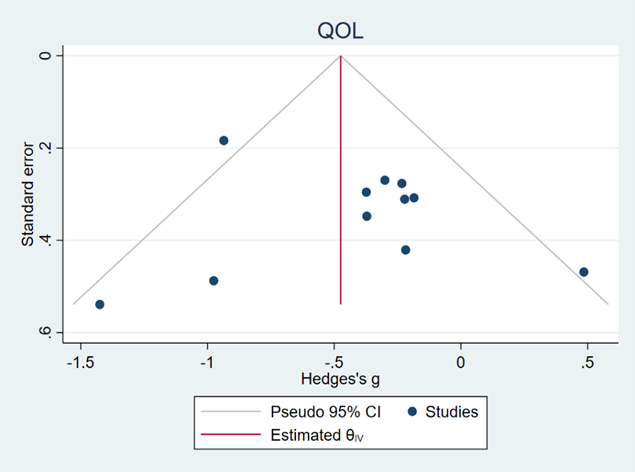


**Figure 5.** Funnel plot to examine publication bias of QoL.

**Figure 7.**


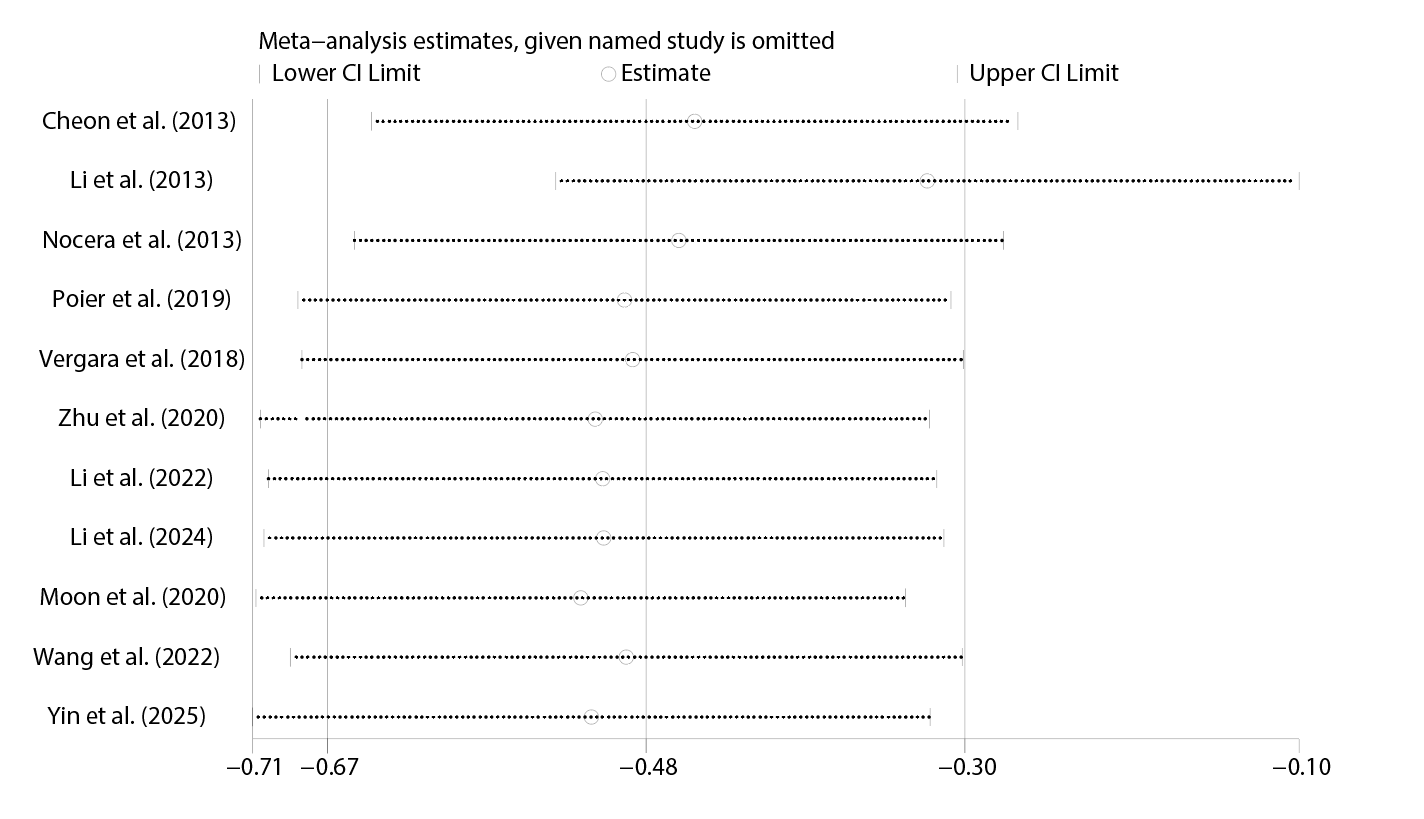

Supplement: Supplementary file 1 [file Data_Sheet_1.docx]
